# AllerGen’s 8th research conference

**DOI:** 10.1186/s13223-016-0164-7

**Published:** 2016-12-08

**Authors:** Marie-Claire Arrieta, Andrea Arevalos, Leah Stiemsma, Marta E. Chico, Carlos Sandoval, Minglian Jin, Jens Walter, Phil Cooper, Brett Finlay, Emilie Bernatchez, Matthew J. Gold, Anick Langlois, Pascale Blais-Lecours, Caroline Duchaine, David Marsolais, Kelly M. McNagny, Marie-Renée Blanchet, Jordan Brubacher, Bimal Chhetri, Kelly Sabaliauskas, Kate Bassil, Jeff Kwong, Frances Coates, Tim K. Takaro, Angela Chow, Gregory E. Miller, Edith Chen, Piushkumar J. Mandhane, Stuart E. Turvey, Susan J. Elliott, Allan B. Becker, Padmaja Subbarao, Malcolm R. Sears, Anita L. Kozyrskyj, Aimée Dubeau, Zihang Lu, Susan Balkovec, Krzysztof Kowalik, Per Gustafsson, Felix Ratjen, Rachel D. Edgar, Nicole R. Bush, Julie L. MacIssac, Lisa M. McEwen, Thomas W. Boyce, Michael S. Kobor, Melanie Emmerson, Aimée Dubeau, Zihang Lu, Bingqing Shen, Krzysztof Kowalik, Felix Ratjen, Theo J. Moraes, Sofianne Gabrielli, Ann Clarke, Harley Eisman, Judy Morris, Lawrence Joseph, Sebastien LaVieille, Moshe Ben-Shoshan, Sumaiya A. Islam, Christof Brückmann, Vanessa Nieratschker, Kyla C. Jamieson, David Proud, Cynthia Kanagaratham, Pierre Camateros, Frantisek Kopriva, Jennifer Henri, Marian Hajduch, Danuta Radzioch, Liane J. Kang, Petya T. Koleva, Catherine J. Field, Tedd Konya, James A. Scott, Theodore Konya, Meghan B. Azad, Jeff Brook, David Guttman, CHILD Study Investigators, Manjeet Kumari, Sarah L. Bridgman, Mon H. Tun, Rupasri Mandal, David S. Wishart, Amy H. Y. Lee, Jeff Xia, Erin Gill, Bob Hancock, Danay Maestre, Darren Sutherland, Jeremy Hirota, Olga Pena, Christopher Carlsten, Lisa M. McEwen, Meaghan J. Jones, Julia L. MacIsaac, William H. Dow, Luis Rosero-Bixby, David H. Rehkopf, Takeshi Morimoto, Steven G. Smith, John-Paul Oliveria, Suzanne Beaudin, Abbey Schlatman, Karen Howie, Caitlin Obminski, Graeme Nusca, Roma Sehmi, Gail M. Gauvreau, Paul M. O’Byrne, Michelle North, Cheng Peng, Marco Sanchez-Guerra, Hyang-Min Byun, Anne K. Ellis, Andrea A. Baccarelli, Joseph O. Okeme, Suman Dhal, Aman Saini, Miriam L. Diamond, Christopher J. Olesovsky, Brittany M. Salter, Michael Wang, Paige Lacy, Michael J. O’Sullivan, Chan Y. Park, Jeffrey J. Fredberg, Anne-Marie Lauzon, James G. Martin, Min Hyung Ryu, Neeloffer Mookherjee, Christopher Carlsten, Elinor Simons, Diana Lefebvre, David Dai, Amrit Singh, Casey P. Shannon, Young Woong Kim, Chen Xi Yang, J. Mark FitzGerald, Louis-Philippe Boulet, Scott J. Tebbutt, Gurpreet K. Singhera, S. JasemineYang, Delbert R. Dorscheid, Hasantha Sinnock, Susan Goruk, Hamid Tavakoli, Larry D. Lynd, Mohsen Sadatsafavi, Mark W. Tenn, Jenny Thiele, Daniel E. Adams, Lisa M. Steacy, Anne K. Ellis, Bahar Torabi, Sarah De Schryver, Duncan Lejtenyi, Ingrid Baerg, Edmond S. Chan, Bruce D. Mazer, Maxwell M. Tran, Wei Hao Dai, Wendy Lou, Radha S. Chari, CHILD Study Investigators, Edward M. Conway, Helen Neighbour, Mark Larché, Scott J Tebbutt

**Affiliations:** 1 Michael Smith Laboratories and Department of Microbiology and Immunology, University of British Columbia, Vancouver, BC Canada; 2 Laboratorio FEPIS-UIDE, Universidad Internacional del Ecuador, Quito, Ecuador; 3 Child and Family Research Institute, University of British Columbia, Vancouver, BC Canada; 4 Department of Agricultural, Food and Nutritional Sciences, University of Alberta, Edmonton, AB Canada; 5 St George’s University of London, London, UK; 6 Department of Biochemistry, University of British Columbia, Vancouver, BC Canada; 7 Centre de recherche de l’Institut universitaire de cardiologie et de pneumologie de Québec, Université Laval, Quebec City, QC Canada; 8 Campbell Family Institute for Breast Cancer Research, Princess Margaret Cancer Centre, Toronto, ON Canada; 9 Biomedical Research Center, University of British Columbia, Vancouver, BC Canada; 10 Faculty of Health Sciences, Simon Fraser University, Burnaby, BC Canada; 11 Toronto Public Health, Toronto, ON Canada; 12 Dalla Lana School of Public Health, University of Toronto, Toronto, ON Canada; 13 Institute for Clinical Evaluative Sciences, Toronto, ON Canada; 14 Aerobiology Research Laboratories, Nepean, ON Canada; 15 Department of Applied Health Science, Indiana University, Bloomington, IN USA; 16 Department of Psychology, Northwestern University, Evanston, IL USA; 17 Division of Pediatric Respirology, Pulmonary and Asthma Department of Pediatrics, University of Alberta, Edmonton, AB Canada; 18 Department of Pediatrics, University of British Columbia, Vancouver, BC Canada; 19 Department of Geography and Environmental Management, University of Waterloo, Waterloo, ON Canada; 20 Section of Allergy and Immunology, Department of Pediatrics and Child Health, University of Manitoba, Winnipeg, MB Canada; 21 Department of Pediatrics, University of Toronto, Toronto, ON Canada; 22 Department of Medicine, Faculty of Health Sciences, McMaster University, Hamilton, ON Canada; 23 Division of Respiratory Medicine, Department of Pediatricsand Program in Physiology and Experimental Medicine, The Research Institute, The Hospital for Sick Children, Toronto, ON Canada; 24 Department of Physiology, Faculty of Medicine, University of Toronto, Toronto, ON Canada; 25 Department of Pediatrics, Central Hospital, Skövde, Sweden; 26 Medical Genetics, The University of British Columbia, Vancouver, BC Canada; 27 Psychiatry, University of California–San Francisco, San Francisco, CA USA; 28 Pediatrics, University of California–San Francisco, San Francisco, CA USA; 29 Faculty of Science, University of Waterloo, Waterloo, ON Canada; 30 Division of Pediatric Allergy and Clinical Immunology, Department of Pediatrics, McGill University Health Center, Montréal, QC Canada; 31 Division of Rheumatology, Department of Medicine, University of Calgary, Calgary, AB Canada; 32 Division of Allergy and Clinical Immunology, Department of Medicine, McGill University Health Center, Montréal, QC Canada; 33 Department of Emergency Medicine, Hôpital du Sacré-Coeur, Montréal, QC Canada; 34 Department of Epidemiology and Biostatistics, McGill University, Montréal, QC Canada; 35 Food DirectorateHealth Canada, Ottawa, ON Canada; 36 Centre for Molecular Medicine and Therapeutics, Child and Family Research Institute, Vancouver, BC Canada; 37 Department of Psychiatry and Psychotherapy, University of Tübingen, Tübingen, Germany; 38 Department of Physiology and Pharmacology, University of Calgary, Calgary, AB Canada; 39 Department of Human Genetics, McGill University, Montréal, QC Canada; 40 Division of Experimental Medicine, Faculty of Medicine, McGill University, Montréal, QC Canada; 41 Department of Medicine, Faculty of Medicine, University of British Columbia, Vancouver, BC Canada; 42 Faculty of Medicine and Dentistry, Institute of Molecular and Translational Medicine, Palacký University, Olomouc, Czech Republic; 43 McGill University Health Center-Research Institute, Montréal, QC Canada; 44 School of Public Health, University of Alberta, Edmonton, AB Canada; 45 Southern Ontario Centre for Atmospheric Aerosol Research, University of Toronto, Toronto, ON Canada; 46 Air Quality Research Division, Environment Canada, Toronto, ON Canada; 47 Department of Pediatrics and Child Health, University of Manitoba and Children’s Hospital Research Institute of Manitoba, Winnipeg, MB Canada; 48 Canadian Healthy Infant Longitudinal Development Study, Hamilton, ON Canada; 49 The Metabolomics Innovation Centre, Edmonton, AB Canada; 50 Centre for Microbial Diseases and Immunity Research, University of British Columbia, Vancouver, BC Canada; 51 Department of Microbiology and Immunology, University of British Columbia, Vancouver, BC Canada; 52 Department of Parasitology, McGill University, Montréal, QC Canada; 53 Wellcome Trust Sanger Institute, Hinxton, UK; 54 Department of Medicine, University of British Columbia, Vancouver, BC Canada; 55 Centro Centroamericano de Población, Universidad de Costa Rica, San José, Costa Rica; 56 Department of Demography, University of California, Berkeley, CA USA; 57 Division of General Medical Disciplines, School of Medicine, Stanford University, Stanford, CA USA; 58 Department Medicine, McMaster University, Hamilton, ON Canada; 59 Laboratory of Environmental Epigenetics, Department of Environmental Health, Harvard T.H. Chan School of Public Health, Boston, MA USA; 60 Institute of Cellular Medicine, Human Nutrition Research Centre, Newcastle University, Newcastle upon Tyne, United Kingdom; 61 Department of Medicine, Allergy Research Unit, Queen’s University Kingston General Hospital, Kingston, ON Canada; 62 Department of Physical and Environmental Science, University of Toronto Scarborough, Toronto, ON Canada; 63 Department of Earth Sciences, University of Toronto, Toronto, ON Canada; 64 Department Medicine, University of Alberta, Edmonton, AB Canada; 65 Department of Physiology, McGill University, Montréal, QC Canada; 66 Department of Environmental Health, Harvard School of Public Health, Boston, MA 02115 USA; 67 Department of Respiratory Medicine, University of British Columbia, Vancouver, BC Canada; 68 Department of Internal Medicine and Immunology, University of Manitoba, Winnipeg, MB Canada; 69 Canadian Respiratory Research Network (CRRN), Ottawa, ON Canada; 70 Division of Respirology, Department of Medicine, McMaster University, Hamilton, ON Canada; 71 Division of Allergy and Immunology, Department of Pediatrics, British Columbia Children’s Hospital, University of British Columbia, Vancouver, BC Canada; 72 Prevention of Organ Failure (PROOF) Centre of Excellence, Vancouver, BC Canada; 73 Centre de Pneumologie de L’Hôpital, Université Laval, Québec City, QC Canada; 74 Centre for Heart Lung Innovation, University of British Columbia, Vancouver, BC Canada; 75 Department of Pediatrics, University of Alberta, Edmonton, AB Canada; 76 Department of Pediatrics, Child and Family Research Institute and BC Children’s Hospital, University of British Columbia, Vancouver, BC Canada; 77 Department of Medicine, Institute for Heart and Lung Health, University of British Columbia, Vancouver, BC Canada; 78 Centre for Clinical Epidemiology and Evaluation, University of British Columbia, Vancouver, BC Canada; 79 Faculty of Pharmaceutical Sciences, University of British Columbia, Vancouver, BC Canada; 80 Department of Biomedical and Molecular Sciences, Queen’s University, Kingston, ON Canada; 81 Allergy Research Unit, Kingston General Hospital, Kingston, ON Canada; 82 The Research Institute of the McGill University Health Centre, Montréal, QC Canada; 83 University of Toronto, Toronto, ON Canada; 84 University of Manitoba, Winnipeg, MB Canada; 85 Department of Pediatrics and Child Health, University of Manitoba, Winnipeg, MB Canada; 86 James Hogg Research Centre for Heart Lung Innovation, St. Paul’s Hospital, University of British Columbia, Vancouver, BC Canada; 87 Centre for Blood Research, University of British Columbia, Vancouver, BC Canada; 88 Division of Respiratory Medicine, Department of Medicine, University of British Columbia, Vancouver, BC Canada; 89 Experimental Medicine, University of British Columbia, Vancouver, BC Canada; 90 Firestone Institute for Respiratory Health, McMaster University, Hamilton, ON Canada

## A1 Early life intestinal microbial alterations are associated with pediatric asthma in rural Ecuador

### Marie-Claire Arrieta^1^, Andrea Arevalos^2^, Leah Stiemsma^3^, Marta E. Chico^2^, Carlos Sandoval^2^, Minglian Jin^4^, Jens Walter^4^, Phil Cooper^2,5^, Brett Finlay^1,6^

#### ^1^Michael Smith Laboratories and Department of Microbiology and Immunology, University of British Columbia, Vancouver, BC, Canada, ^2^Laboratorio FEPIS-UIDE, Universidad Internacional del Ecuador, Quito, Ecuador, ^3^Child and Family Research Institute, University of British Columbia, Vancouver, BC, Canada, ^4^Department of Agricultural, Food and Nutritional Sciences, University of Alberta, Edmonton, AB, Canada, ^5^St George’s University of London, London, UK, ^6^Department of Biochemistry, University of British Columbia, Vancouver, BC, Canada

##### **Correspondence:** Marie-Claire Arrieta - marrieta@msl.ubc.ca

*Allergy, Asthma & Clinical Immunology* 2016, **12(Suppl 2)**:A1

**Background:** Asthma is the most prevalent chronic disease among children and affects 235 million people worldwide [1]. Although the incidence of asthma in South America is among the highest worldwide, underlying causes and disease phenotypes are poorly defined and may differ to developed countries. Recent evidence in mice [2] and human [3] has identified a ‘critical window’ early in life where the effects of gut microbial changes (dysbiosis) are most influential in immune development and experimental asthma. Given the differences in gut microbiota between North and South American populations, we aimed to validate our previous work in a microbially-different human population.

**Methods:** We compared the gut microbiota of 97 infants from the coastal community Las Esmeraldas, Ecuador at 3 months of age by 16S sequencing (V4 region) Illumina MiSeq. Subjects were grouped into atopic-wheezers (n = 27) and controls (n = 70) based on a skin prick test and wheezing history at 1 year of age. An exact logistic regression model was developed to evaluate the risk associated with the AW group according to specific clinical and epidemiological metadata. Metagenomes were predicted from 16S rRNA OTU data using PICRUSt, and categorized by function using KEGG Orthology. The concentration of fecal short chain fatty acids (SCFA) was determined by gas chromatography.

**Results**: Atopy and wheezing at 1 year of age was significantly associated with asthma diagnosis at 5 years (OR 17.8), birth by C-section (OR 3.1), potable water at home (OR 2.7) and in utero exposure to antibiotics (OR 2.9). This phenotype was also significantly associated with eosinophil concentration at 2 and 5 years (p < 0.05), number of episodes of respiratory infections (p < 0.01), maternal load with *Trichuris trichiura* during pregnancy (p < 0.05), and inversely associated with the number of diarrheal episodes by 1 year of age (p < 0.05). Similar to what we had previously found in Canadian babies, atopic-wheezing Ecuadorian babies also exhibit gut microbial dysbiosis at 3 months of age. However, the microbial alterations were different and more pronounced in Ecuadorian babies. Predicted metagenomic analysis showed significant differences in genes involved in carbohydrate and taurine metabolism. Fecal acetate was significantly reduced in atopic wheezers. Ongoing experiments will determine if differences in eukaryome are also associated with asthma risk in this population.

**Conclusions:** This study further supports the importance of the microbiota during the first 100 days of life, although the characteristics of the microbial dysbiosis and epidemiological associations depend on geographical location. Reduced fecal acetate as a common feature in both populations suggests that different microbial alterations may have similar metabolic outcomes.

**References**

1. Asthma. Geneva: World Health Organization; 2011.

2. Russell SL, Gold MJ, Hartmann M, Willing BP, Thorson L, Wlodarska M, et al. Early life antibiotic-driven changes in microbiota enhance susceptibility to allergic asthma. EMBO Rep. 2012;13:440–7.

3. Arrieta MC, Stiemsma LT, Dimitriu PA, Thorson L, Russell S, Yurist-Doutsch S, et al. Early infancy microbial and metabolic alterations affect risk of childhood asthma. Sci Transl Med. 2015;7:307ra152.

## A2 Cellular mechanisms involved in the allergic lung response to the environmental bioaerosol archaea *Methanosphaera stadtmanae*

### Emilie Bernatchez^1^, Matthew J. Gold^2^, Anick Langlois^1^, Pascale Blais-Lecours^1^, Caroline Duchaine^1^, David Marsolais^1^, Kelly M. McNagny^3^, Marie-Renée Blanchet^1^

#### ^1^Centre de recherche de l’Institut universitaire de cardiologie et de pneumologie de Québec, Université Laval, Québec City, QC, Canada, ^2^Campbell Family Institute for Breast Cancer Research, Princess Margaret Cancer Centre, Toronto, ON, Canada, ^3^Biomedical Research Center, University of British Columbia, Vancouver, BC, Canada

##### **Correspondence:** Emilie Bernatchez - emilie.bernatchez@criucpq.ulaval.ca

*Allergy, Asthma & Clinical Immunology* 2016, **12(Suppl 2)**:A2

**Background:** Bioaerosols in occupational environments are associated with the development of inflammatory lung diseases [1]. The archaea specie *Methanosphaera stadtmanae* (MSS) is found in high concentrations in poultries, dairy farms and swine confinement buildings bioaerosols (up to 10^8^ archaea/m^3^) [2–4]. In mice, MSS induces an inflammatory lung response characterized by T cells, eosinophils, neutrophils, and IgG production [5]. However, in order to better understand the potential impact of MSS exposure on the pulmonary health of workers, further characterization of the immunopathology induced by this archaeon is essential.

**Methods:** Wild-type mice (WT) were exposed to either 3 μg (low dose) or 100 μg (high dose) MSS by intranasal instillation, three consecutive days a week for 3 weeks. Four days after last exposure, mice were euthanized. The immune response polarity was determined by flow cytometry analysis of T_H_ cell populations (T_H_1, T_H_2 and T_H_17) and by the evaluation of antigen-specific serum immunoglobulins. Lung airway hyperresponsiveness was measured using a Flexivent apparatus. To study possible mechanisms involved in the response, inflammation severity was analyzed in ILC2-, mast cell-, eosinophil-deficient, *Cd34* ^−*/*−^ and *Tlr4* ^−*/*−^ mice exposed to MSS using broncho-alveolar lavages cell counts.

**Results:** In WT mice, 3 μg MSS induced a weak T_H_2 response and a strong T_H_17 lung response, characterized by IgG_1_ (but not IgG_2a_ and IgE) production. Interestingly, high doses of MSS led to a lower eosinophil counts and full polarization into a T_H_17 response. Moreover, at low MSS dose, mice did not develop airway hyperresponsiveness. Using transgenic mice (eosinophil- and mast cell-deficient), we found that eosinophils, mast cells, and ILC2s cells are not required for the inflammatory response to MSS. No differences were found in *Cd34* ^−*/*−^ mice, indicating no important role for this molecule in the response. *Tlr4* ^−*/*−^ mice exposed to MSS had a strong decrease in airway inflammation compared to WT mice. However, as we found endotoxins in our MSS lot, part of the phenotype observed in *Tlr4* ^−*/*−^ mice could be attributable to endotoxin activity.

**Conclusions:** We conclude that MSS exposure induces a T_H_17-dominated inflammatory lung response which could be harmful to workers exposed to environments where high levels of MSS and endotoxin are present in bioaerosols.

**Acknowledgements:** This work was supported by Institut de recherche Robert-Sauvé en santé et sécurité du travail (IRSST) and AllerGen NCE Inc., a member of the Networks of Centres of Excellence Canada program. We thank Dr. John Schrader for the *Tlr4* ^−*/*−^ mice.

**References**

1. Department of Health and Human Services, C.f.D.C.a.P., National Institute for Occupational Safety and Health. Respiratory disease in agricultural workers: mortality and morbidity statistics. DHHS (NIOSH). 2007:2007–106.

2. Blais Lecours P, et al. Characterization of bioaerosols from dairy barns: reconstructing the puzzle of occupational respiratory diseases by using molecular approaches. Appl Environ Microbiol. 2012;78(9):3242–8.

3. Just N, et al. Archaeal characterization of bioaerosols from cage-housed and floor-housed poultry operations. Can J Microbiol. 2013;59(1):46–50.

4. Nehme B, et al. Culture-independent characterization of archaeal biodiversity in swine confinement building bioaerosols. Appl Environ Microbiol. 2009;75(17):5445–50.

5. Blais Lecours P, et al. Immunogenic properties of archaeal species found in bioaerosols. PLoS One. 2011;6(8):e23326.

## A3 Climate change, asthma and allergy risk in Toronto

### Jordan Brubacher^1^, Bimal Chhetri^1^, Kelly Sabaliauskas^2^, Kate Bassil^2,3^, Jeff Kwong^4^, Frances Coates^5^, Tim Takaro^1^

#### ^1^Faculty of Health Sciences, Simon Fraser University, Burnaby, British Columbia, Canada, ^2^Toronto Public Health, Toronto, ON, Canada, ^3^Dalla Lana School of Public Health, University of Toronto, Toronto, ON, Canada, ^4^Institute for Clinical Evaluative Sciences, Toronto, ON, Canada, ^5^Aerobiology Research Laboratories, Nepean, ON, Canada

##### **Correspondence:** Jordan Brubacher - jbrubach@sfu.ca

*Allergy, Asthma & Clinical Immunology* 2016, **12(Suppl 2)**:A3

**Background:** Plant pollen and fungal spores of select species contribute to asthma and allergic exacerbations and may cause these chronic illnesses to develop [1]. These organisms interact with the climate to influence production, timing and dispersal of their airborne glycol-proteins. Using retrospective data, we describe associations among pollen, spores, allergies and asthma in Toronto, Canada as a first step towards gaining a better understanding of future trends in a changing climate.

**Methods:** Time-series analyses were used to measure associations between health encounters, aeroallergens and air pollutants from March 2004 to November 2014. Weekly allergic rhinitis (AR) and asthma were modeled as a function of weekly aeroallergens and air pollutants using a negative binomial generalized linear model adjusted for demographics and seasonality. Population was used as an offset. Weekly health outcome data were defined using International Classification of Disease and Ontario Health Insurance Plan codes for AR and asthma.

**Results:** Health data indicated 1,421,822 AR and 1,640,674 asthma encounters for the metropolitan population of 2.8 million during the study period. Preliminary regression results for AR included positive linear associations with weed pollen, grass pollen, basidiomycota spores and nitrogen dioxide (NO_2_) and a quadratic association with deciduous tree pollen (Table 1). Asthma results included positive linear associations with deciduous tree pollen, grass pollen and deuteromycota spores and a quadratic association with NO_2_.

**Table 1:** Preliminary multivariable regression results (negative binomial generalized linear model)

Model	Variable	Coefficient	95% Confidence intervals		p
Allergies	Deciduous trees	0.0300	0.0220	0.0379	<0.001
	(Deciduous trees)^2^	−0.000002	−0.000003	−0.000001	<0.001
	Weeds	0.0523	0.0330	0.0719	<0.001
	Grasses	0.1004	0.0393	0.1622	0.001
	Basidiomycota	0.0016	0.0006	0.0027	0.003
	NO_2_	1.0808	0.4739	1.6894	<0.001
Asthma	Deciduous trees	0.0044	0.0017	0.0071	0.001
	Grasses	0.0670	0.0170	0.1176	0.008
	Deuteromycota	0.0009	0.0005	0.0014	<0.001
	NO_2_	5.8351	3.2237	8.4242	<0.001
	(NO_2_)^2^	−0.0990	−0.1730	−0.0230	0.010

All estimates are exponentiated

Estimates for pollen and spores are per 100 grains and for NO_2_ is per part per billion

**Conclusion:** Preliminary findings suggest that pollen from deciduous trees, weeds, and grasses and basidiomycota spores are contributing to the AR burden in Toronto, while the asthma burden is associated with deciduous tree and grass pollen and deuteromycota spores. Both AR and asthma appear to be associated with ambient NO_2_ levels. Disentangling the effects of aeroallergens from other environmental factors like weather and air pollution are necessary to identify taxa responsible for the largest burden, and to subsequently investigate what impacts climate change may have on the duration, timing and intensity of their individual pollen seasons. The findings of this research will contribute to the risk assessment of climate and health vulnerabilities undertaken by the City of Toronto. We hope to identify opportunities for illness prevention as pollen seasons may be extended and precipitation and temperature patterns for fungal ecology shift with climate change.

**Acknowledgements:** Institute for Clinical Evaluative Sciences Data and Analytic Services, Toronto, ON, Canada

**Reference**

1. D’Amato G, Holgate S, Pawankar R, Ledford D, Cecchi L, Al-Ahmad M, Annesi-Maesano I. Meteorological conditions, climate change, new emerging factors, and asthma and related allergic disorders. A statement of the World Allergy Organization. World Allergy Organ J. 2015;8(1):1.

## A4 Distinct trajectories in depressive symptoms and perceived stress from pregnancy to the postnatal period

### Angela Chow^1^, Gregory E. Miller^2^, Edith Chen^2^, Piushkumar J. Mandhane^3^, Stuart E. Turvey^4^, Susan J. Elliott^5^, Allan B. Becker^6^, Padmaja Subbarao^7^, Malcolm R. Sears^8^, Anita L. Kozyrskyj^4^

#### ^1^Department of Applied Health Science, Indiana University, Bloomington, IN, USA, ^2^Department of Psychology, Northwestern University, Evanston, IL, USA, ^3^Department of Pediatrics, University of Alberta, Edmonton, AB, Canada, ^4^Department of Pediatrics, University of British Columbia, Vancouver, BC, Canada, ^5^Department of Geography and Environmental Management, University of Waterloo, Waterloo, ON, Canada, ^6^Department of Pediatrics & Child Health, University of Manitoba, Winnipeg, MB, Canada, ^7^Department of Pediatrics, University of Toronto, Toronto, ON, Canada, ^8^Department of Medicine, McMaster University, Hamilton, ON, Canada

##### **Correspondence:** Angela Chow - chowa@indiana.edu

*Allergy, Asthma & Clinical Immunology* 2016, **12(Suppl 2)**:A4

**Background:** Despite recent interest [1], little is known about the determinants of maternal stress and depression trajectories from pregnancy to the postnatal period in Canadian society. This study aimed to: (1) identify distinct trajectories of pre/postnatal maternal perceived stress and depressive symptoms, (2) assess longitudinal associations between these two maternal distress constructs and (3) test associations between maternal distress trajectories and sociodemographic characteristics, such as immigration status.

**Methods:** The sample consists of 3307 women participating in the Canadian Healthy Infant Longitudinal Development (CHILD) Study. Data on depressive symptoms (using Center for Epidemiologic Studies Depression Scale) and perceived stress (using Perceived Stress Scale) were collected at recruitment (mean = 27 weeks) and at 36 weeks of gestation in the prenatal period, and then at 6 months, 1, 1.5, and 2 years in the postnatal period.

Data on maternal demographics, immigration status, depressive history and employment were collected at recruitment. General growth mixture models were conducted in Mplus 7.3 to identify distinct trajectories in depressive symptoms and perceived stress. Configural frequency analysis examined the associations between trajectory memberships. Multinomial regression was used to test how trajectory group memberships were related to maternal sociodemographics, such as immigration status and employment.

**Results:** Five trajectory patterns for depressive symptoms (*chronic*: 2.2%; *antepartum*: 5.6%; *postpartum*: 7.6%, *never*-*higher scores:* 27.7%, *never*-*low score*s: 56.9%) and five trajectory patterns for perceived stress (*chronic*: 6.9%; *antepartum*: 6.3%; *postpartum*: 22.6%, *never*-*higher scores*: 40.2%, *never*-*low scores*: 24%) were found. Chronic stress was significantly associated with chronic depression or depression in the antepartum or postpartum period. Women with elevated levels of depressive symptoms in the antepartum period were at significant risk of higher stress across pregnancy and the first 2 years after delivery. Also, women from the two never stressed groups were significantly unlikely to suffer from depression at any point of the studied period. New immigrant women who lived in Canada for 6–10 years but not new comers who moved to Canada within the last five years, were at a higher risk for chronic and postpartum depression.

**Conclusions:** This study highlights the extent of stress and depression among Canadian women during a critical time period for infant development. It also provides evidence for the “healthy immigrant effect” for recent immigrants but points to an elevated risk for depression during pregnancy and the postnatal period for immigrant women who have settled in their new homeland for 5–10 years.

**Acknowledgements:** Support was provided by CIHR and AllerGen NCE. We would like to acknowledge the participants and investigators of the CHILD Study.

**Reference**

1. Luoma I, Korhonen M, Salmelin RK, Helminen M, Tamminen T. Long-term trajectories of maternal depressive symptoms and their antenatal predictors. J Affect Disord. 2015;170:30–38.

## A5 The effects of birth weight on infant pulmonary function

### Aimée Dubeau^1^, Zihang Lu^1^, Susan Balkovec^1^, Krzysztof Kowalik^1,2^, Per Gustafsson^3^, Felix Ratjen^1^, Malcolm R. Sears^4^, and Padmaja Subbarao^1,2^

#### ^1^Division of Respiratory Medicine, Department of Pediatrics, and Program in Physiology and Experimental Medicine, The Research Institute, The Hospital for Sick Children, Toronto, ON, Canada, ^2^Department of Physiology, Faculty of Medicine, University of Toronto, Toronto, ON, Canada, ^3^Department of Pediatrics, Central Hospital, Skövde, Sweden, ^4^Department of Medicine, McMaster University, Hamilton, ON, Canada

##### **Correspondence:** Aimée Dubeau - aimee.dubeau@sickkids.ca

*Allergy, Asthma & Clinical Immunology* 2016, **12(Suppl 2)**:A5

**Background**: Given the importance of the prenatal environment on organogenesis [1], we hypothesized that birth weight as a manifestation of the prenatal environment may have impacts on infant lung function beyond the initial measurement and may play a role in long-term respiratory health. In this study, we have examined the impacts of birth weight on infant lung function in a sub-cohort of a general population birth cohort study, the Canadian Healthy Infant Longitudinal Development (CHILD) Study to further understand the role of prenatal environment on lung health.

**Methods:** Pregnant women recruited to the Toronto site of the CHILD Study were invited to participate in an infant lung function study. Pulmonary function tests included multiple breath washout (MBW) testing, from which we derived the lung clearance index (LCI), performed on three occasions, early, mid and late infancy. To understand the role of the birth weight, infants with weights in the upper centile (>90th, i.e. 4000 g) and lowest centile (<10th, i.e. 2800 g) were compared to those within these centiles. Linear mixed effects models with random intercept were used to assess the effects of birth weight on lung function measurements.

**Fig. 1:**
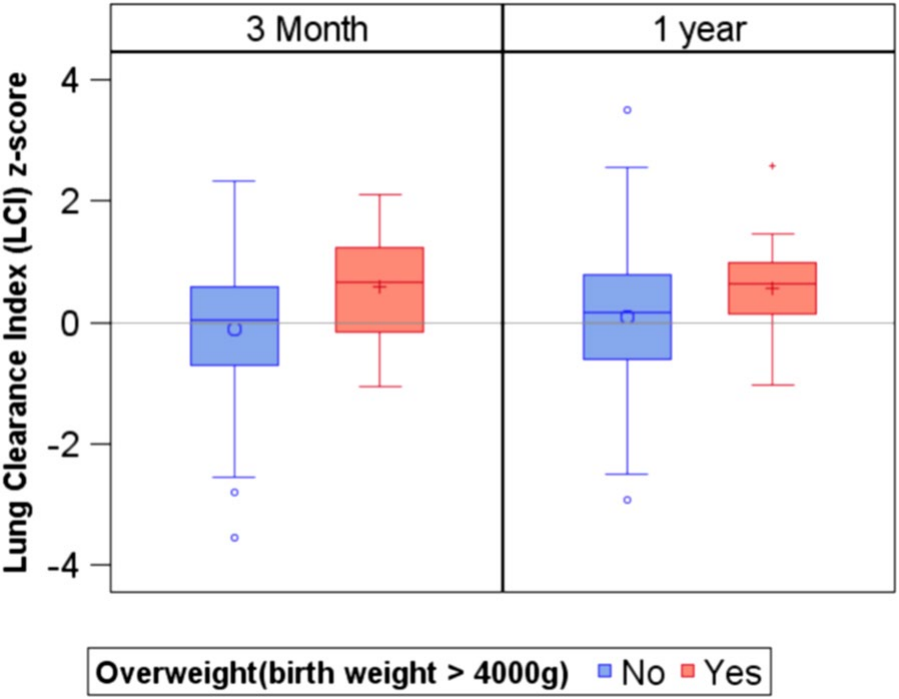
Relationship between birth weight and LCI measured at 3 months and 1 year

**Results:** We obtained pulmonary function values from 218 (30% of the total Toronto cohort) participants (58% males and 42% females) during infancy, of which 32 completed one visit, 106 completed two visits and 80 completed three visits. Data from all visits were included in analysis. These participants were on average (standard deviation) 9.07 (3.92) months old at their first visit, and had length-for-age z-score and weight-for-age z-score of −0.28 (1.24) and 0.02 (0.99), respectively. Twenty-five (11%) of participants were classified as overweight and 18 (8%) of participants were classified as underweight at birth. The LCI was on average (Standard Error) 0.26 (0.11) units (p = 0.02) higher in those infants that were born overweight, after adjusting for age, height and gender (Fig. 1). No effect of weight <10% percentile on LCI was observed. This trend is consistent when using LCI z-score as the outcome, with 0.56 (standard error = 0.2, p = 0.006) units higher in those overweight infants.

**Conclusions:** The prenatal environment, as manifested by birth weight, has a significant influence on lung function, as measured by LCI, in the first year of life.

**Acknowledgements:** This work was supported by the Canadian Institute for Health Research (CIHR), the SickKids Foundation, a private donation from Debbie and Donald Morrison and the AllerGen NCE Inc.

**Reference**

1. Harding R, Maritz G. Maternal and fetal origins of lung disease in adulthood. Semin Fetal Neonatal Med. 2012;17(2):67–72.

## A6 Immune genes are differentially methylated in relation to early life stress exposure

### Rachel D. Edgar^1^, Nicole R. Bush^2,3^, Julie L. MacIssac^1^, Lisa M. McEwen^1^, Thomas W. Boyce^2,3^, Michael S. Kobor^1^

#### ^1^Medical Genetics, The University of British Columbia, Vancouver, BC, Canada, ^2^Psychiatry, University of California–San Francisco, San Francisco, CA, USA, ^3^Pediatrics, University of California–San Francisco, San Francisco, CA, USA

##### **Correspondence:** Rachel D. Edgar - redgar@cmmt.ubc.ca

*Allergy, Asthma & Clinical Immunology* 2016, **12(Suppl 2)**:A6

**Background**: Changes in an individual’s immune system in response early life stress have important implications for the lifetime health of an individual. One potential mechanism through which early life stress could become biologically embedded to effect immune function is epigenetics. Here we have performed an epigenome-wide association study on the effects of early life stress and adversity measures on DNA methylation, to examine which genomic pathways show differential methylation.

**Methods**: DNA methylation was interrogated with the Illumina Infinium HumanMethylation450 BeadChip [1] and genotyping was done on the Psych Chip [2]. Genetic ancestry was called using identical by shared state (IBS) clustering in the statistical program Plink (version 1.07) [3]. DNA methylation analysis was performed on all probes on the array, using linear mixed effects modeling. Enrichment of Gene Ontology terms in the list of differentially methylated genes was tested using overrepresentation analysis was done ErmineJ [4].

**Results**: In an epigenome-wide analysis of the effects of early life stress we found genes involved immune related pathways were enriched for differential methylation.

**Conclusions**: We have seen that early life adversity embeds a distinct signal on the epigenome based on the source of the early life stress. However, multiple forms of stress we have examined share a signal of differential methylation in immune genes.

**Acknowledgements:** This research was supported by AllerGen NCE Inc. (the Allergy, Genes and Environment Network), a member of the Networks of Centres of Excellence Canada program, the National Institute of Mental Health, by funding from Robert Wood Johnson Health and Society Scholar program via the Health Disparities Working Group at UCSF, the UC Berkeley Population Center, the R. Howard Webster Foundation, and the Brain Canada Foundation.

**References**

1. Bibikova M, Barnes B, Tsan C, Ho V, Klotzle B, Le JM, et al. High density DNA methylation array with single CpG site resolution. Genomics. 2011;98(4):288–95.

2. Psychiatric Genomics Consortium—Psych Chip. http://www.med.unc.edu/pgc/psychchip.

3. Purcell S, Neale B, Todd-Brown K, Thomas L, Ferreira MAR, Bender D, et al. PLINK: a tool set for whole-genome association and population-based linkage analyses. Am J Hum Genet. 2007;81(3):559–75.

4. Gillis J, Mistry M, Pavlidis P. Gene function analysis in complex data sets using ErmineJ. Nat Protoc. 2010;5(6):1148–59.

## A7 Lung clearance index (LCI) in 3 year old children with clinically assessed preschool asthma

### Melanie Emmerson^1,2^, Aimée Dubeau^2^, Zihang Lu^2^, Bingqing Shen^2^, Krzysztof Kowalik^2,3^, Per Gustafsson^4^, Felix Ratjen^2^, Malcolm R. Sears^5^, Theo J. Moraes^2^, Padmaja Subbarao^2,3^

#### ^1^Faculty of Science, University of Waterloo, Waterloo, ON, Canada, ^2^Division of Respiratory Medicine, Department of Pediatrics, and Program in Physiology and Experimental Medicine, The Research Institute, The Hospital for Sick Children, Toronto, ON, Canada, ^3^Department of Physiology, Faculty of Medicine, University of Toronto, ^4^Department of Pediatrics, Central Hospital, Skövde, Sweden, ^5^Department of Medicine, Faculty of Health Sciences, McMaster University, Hamilton, ON, Canada

##### **Correspondence:** Melanie Emmerson - melanie.emmerson@sickkids.ca

*Allergy, Asthma & Clinical Immunology* 2016, **12(Suppl 2)**:A7

**Background:** Asthma diagnosis in preschool children is complicated by its phenotypic overlap with common transient wheezing. As a result, there is an emphasis on predicting the persistence of symptoms into school age. Clinical predictions currently employ models limited by modest sensitivity [1], namely the asthma predictive index (API), and may benefit from the addition of objective lung function measures. The lung clearance index (LCI) is a measure of ventilation inhomogeneity, derived from multiple breath washout (MBW) tests [2]. Previous data has correlated elevated LCI with persistent asthma and multi-trigger wheeze [3]. However, no data exists for LCI in mild asthmatics from the general community. Here, we report LCI measurements from the Canadian Healthy Infant Longitudinal Development (CHILD) study, a general population birth cohort.

**Methods:** Participant data was obtained from the CHILD study Toronto general cohort. During 3-year visits, a subset of participants performed MBW SF_6_ testing, as previously described [4]. Quality control was completed per ATS/ERS guidelines [5]. LCI values were included for healthy controls (HC) and all definite and possible (D&P) preschool asthmatics, as defined by clinical assessment. The HC group had confirmed absence of respiratory symptoms, congenital respiratory problems, and parental smoke exposure. Population demographics explored sex, age, height, and weight, with applicable z-score calculations. Parametric t-tests and Wilcoxon rank sum tests were used to compare mean LCI measurements between groups.

**Results:** At the 3-year visit, acceptable LCI measurements were produced for 101 participants, of which 67 were HC and 34 had definite (n = 6) or possible (n = 28) asthma. Mean LCI (standard deviation) was 6.07 (0.43) and 6.42 (0.78) for HC and D&P asthma, respectively. LCI was an average of 0.35 (95% CI [0.06, 0.63]) units higher in D&P asthma than in healthy controls (p = 0.02). 3 definite and 6 possible asthmatics had an abnormal LCI (defined > 95th percentile). API positivity was not helpful in predicting an abnormal LCI (p = 0.67). We were unable to detect a significant difference in LCI between total API positive (n = 9) and API negative (n = 25) asthmatics (p = 0.188).

**Conclusions:** LCI is elevated in 3-year old children with clinically assessed D&P asthma compared to healthy controls. Future longitudinal studies will determine whether elevated LCI is predictive of persistent asthma at 5 years.

**Acknowledgements:** This work was supported by the Canadian Institute for Health Research (CIHR), the SickKids Foundation, a private donation from Debbie and Donald Morrison and AllerGen NCE Inc.

**References**

1. Luo G, Nkoy F, Stone B, Schmick D, Johnson M. A systematic review of predictive models for asthma development in children. BMC Med Inform Decis Mak. 2015;15(99):1–16.

2. Jensen R, Stanojevic S, Gibney K, Salazar J, Gustafsson P, Subbarao P, Hartl D. Multiple breath nitrogen washout: a feasible alternative to mass spectrometry. PLoS ONE. 2013:E56868.

3. Sonnappa S, Bastardo C, Wade A, Saglani S, McKenzie S, Bush A, Aurora P. Symptom-pattern phenotype and pulmonary function in preschool wheezers. J Allergy Clin Immunol. 2010; 126(3):519–26.

4. Gustafsson PM, Aurora P, Lindblad A. Evaluation of ventilation maldistribution as an early indicator of lung disease in children with cystic fibrosis. Eur Respir J. 2003;22:972–79.

5. Robinson PD, Latzin P, Verbanck S, Hall GL, Horsley A, Gappa M, et al. Consensus statement for inert gas washout measurement using multiple- and single- breath tests. Eur Respir J. 2013;41:507–522.

## A8 Increased drug-induced anaphylaxis visits and factors affecting reaction severity: a 3-year follow-up study in two Emergency Departments in Montréal

### Sofianne Gabrielli^1^, Ann Clarke^2^, Harley Eisman^3^, Judy Morris^4^, Lawrence Joseph^5^, Sebastien LaVieille^6^, Moshe Ben-Shoshan^1^

#### ^1^Division of Pediatric Allergy and Clinical Immunology, Department of Pediatrics, McGill University Health Center, Montréal, QC, Canada, ^2^Division of Rheumatology, Department of Medicine, University of Calgary, Calgary, AB, Canada, ^3^Division of Allergy and Clinical Immunology, Department of Medicine, McGill University Health Center, Montréal, QC, Canada, ^4^Department of Emergency Medicine, Hôpital du Sacré-Coeur, Montréal, QC, Canada, ^5^Department of Epidemiology and Biostatistics, McGill University, Montréal, QC, Canada, ^6^Food Directorate, Health Canada, Ottawa, ON, Canada

##### **Correspondence:** Sofianne Gabrielli - sofiannegabrielli@gmail.com

*Allergy, Asthma & Clinical Immunology* 2016, **12(Suppl 2)**:A8

**Background:** While previous studies have suggested increased rates of anaphylaxis, it is not clear if the rate of drug-induced anaphylaxis is increasing [1, 2]. We aimed to assess the percentage of drug-induced anaphylaxis cases among all Emergency Department (ED) visits due to anaphylaxis in a pediatric and an adult ED in Montréal and to determine factors associated with severe reactions.

**Methods:** Over a 3-year period, children and adults presenting to the Montréal Children’s Hospital and Hôpital du Sacré-Coeur ED with anaphylaxis were recruited as part of the Cross-Canada Anaphylaxis Registry (C-CARE). A standardized data entry form documenting symptoms and triggers of anaphylaxis was collected by the physician. Patients were contacted every 12 months to inquire if the anaphylaxis drug trigger was confirmed. Multivariate logistic regression was used to estimate factors associated with reaction severity.

**Results:** From June 2012 to May 2015, 29 patients presented to the pediatric ED with drug-induced anaphylaxis of which 37.9% were male with a median age of 9.67 years (IQR: 4.6, 15.6). Of these reactions, 6.9% (95% CI 0, 16.7) were classified as severe and 34.5% (95% CI 16.1, 52.9) of patients reacted to antibiotics (Table 2). More than half the patients [58.6% (95% CI 39.6, 77.7)] were treated with epinephrine. At the adult ED, 52 patients presented with anaphylaxis to drugs of which 25.0% were male with a median age of 49.1 years (IQR: 38.0, 62.9). 17.3% (95% CI 6.7, 27.9) of the reactions were severe and 59.6% (95% CI 45.8, 73.4) of patients reacted to antibiotics (Table 2). About half of the patients [48.1% (95% CI 34.0, 62.1)] were treated with epinephrine. The percentage of drug-induced anaphylaxis cases at the Montreal Children’s Hospital and Hôpital du Sacré-Coeur demonstrated a raw increase from year 1 to 3 of 13.8% (95% CI 0.77, 65.9) and 9.62% (95% CI 2.56, 47.4), respectively.

**Table 2:** Characteristics of patients presenting to the Emergency Department with Drug-Induced Anaphylaxis

Variable (%, 95% CI)	Montreal Children’s Hospital	Hôpital Sacré-Coeur
N	29	52
AAR (median, IQR)	9.67 (4.6, 15.6)	49.1 (38.0, 62.9)
Sex (% males)	37.9 (19.1, 56.7)	25.0 (12.8, 37.2)
Cases per year		
2012–2013	27.6 (10.3, 44.9)	28.8 (16.1, 41.6)
2013–2014	31.0 (13.1, 48.9)	32.7 (19.5, 45.9)
2014–2015	41.4 (22.3, 60.4)	38.5 (24.8, 52.1)
Medication type		
Antibiotics	34.5 (16.1, 52.9)	59.6 (45.8, 73.4)
Non-antibiotic drugs	65.5 (47.1, 83.9)	40.4 (26.6, 54.2)
Known drug allergy	6.9 (0, 16.7)	30.6 (17.2, 44.0)
Known food allergy	20.7 (5.0, 36.4)	12.2 (2.7, 21.8)
Known asthma	24.1 (7.6, 40.7)	4.1 (0, 9.8)
Reaction type		
Mild^a^	13.8 (0.4, 27.1)	0
Moderate^b^	79.3 (63.6, 95.0)	82.7 (72.0, 93.3)
Severe^c^	6.9 (0, 16.7)	17.3 (6.7, 27.9)
Treatment in ED		
Epinephrine	58.6 (39.6, 77.7)	48.1 (34.0, 62.1)
Antihistamines	41.4 (22.3, 60.4)	78.8 (67.4, 90.3)
Steroids	17.2 (2.6, 31.9)	86.5 (76.9, 96.1)

^a^Symptoms include urticaria, erythema, angioedema, oral pruritus, nausea, nasal congestion, sneezing, rhinorrhea or throat tightness

^b^Symptoms include crampy abdominal pain, diarrhea, recurrent vomiting, dyspnea, stridor, cough, wheeze, or “light-headedness”

^c^Symptoms include cyanosis, hypoxia, respiratory arrest, hypotension, dysrhythmia, confusion, or loss of consciousness

At the pediatric ED, severe reactions to drugs were associated with parenteral contact route and hospital admittance [OR 1.41 (95% CI 1.16, 1.72) and 1.41 (95% CI 1.05, 1.89)]. At the adult ED, severe reactions to antibiotics were associated with fluoroquinolones and hospital admittance [OR 1.29 (95% CI 1.04, 1.58) and 1.49 (95% CI 1.06, 2.09)].

**Conclusion:** Caregivers should be aware of the risk of severe reactions in adults treated with fluoroquinolones and those requiring IV treatment. Given the increased percentage of ED visits and that 50% of reactions in children and adults were not treated with epinephrine, educational programs prompting the use of epinephrine in drug-induced anaphylaxis are required.

**Acknowledgements:** The study was funded by AllerGen NCE (the Allergy, Genes, and Environment Network) and Health Canada.

**References**

1. Hochstadter et al. C-CARE: comparing two years of anaphylaxis in children treated at the Montreal Children’s Hospital. Allergy, Asthma Clin Immunol. 2014;10(Suppl 1):A6.

2. Asai et al. Rate, triggers, severity and management of anaphylaxis in adults Treated in a Canadian Emergency Department. Int Arch Allergy Immunol. 2014;164:246–52.

## A9 Longitudinal measures of DNA methylation associated with alcohol exposure cessation in purified T-lymphocytes

### Sumaiya A. Islam^1,2†^, Christof Brückmann^3†^, Michael S. Kobor^1,2^, Vanessa Nieratschker^3^

#### ^1^Department of Medical Genetics, University of British Columbia, Vancouver, BC, Canada, ^2^Child and Family Research Institute, Centre for Molecular Medicine and Therapeutics, Vancouver, B C, Canada, ^3^Department of Psychiatry and Psychotherapy, University of Tübingen, Tübingen, Germany

##### **Correspondence:** Sumaiya A. Islam - sislam@cmmt.ubc.ca

^†^Authors contributed equally to this work

*Allergy, Asthma & Clinical Immunology* 2016, **12(Suppl 2)**:A9

**Background:** Previous studies have demonstrated that lifestyle factors such as cigarette smoking and heavy alcohol consumption are associated with differential DNA methylation in peripheral blood [1–3]. However, examination of intervention-based, longitudinal measures of these effects remain limited [4]. Here we aimed to assess genome-wide differences in DNA methylation following an intervention-based cessation of alcohol exposure in purified T-lymphocytes.

**Methods:** Twenty-four Caucasian men were recruited for a 3 week intervention study involving a cessation of excessive alcohol consumption. Magnetic bead separation was used to isolate CD3-positive T-lymphocytes from pre- and post-intervention peripheral blood mononuclear cell (PBMC) samples of participants, as well as 23 healthy male controls that were matched for age, ethnicity and smoking behaviour. DNA methylation was measured at over 450,000 CpG sites genome-wide in these samples using the Illumina HumanMethylation450K Beadchip array. DNA methylation data was subjected to quality control processing, normalization and correction for technical variation. Principal component analysis (PCA) was used to investigate associations to global DNA methylation patterns and linear regression analyses were used to identify site-specific DNA methylation alterations.

**Results:** Smoking (measured as cigarettes per day) and alcohol abuse status were associated with top-ranking principal components, collectively comprising ~20% of the variance in the DNA methylation data. Linear regression analyses with paired testing identified site-specific DNA methylation differences between pre- and post-intervention samples. Interestingly, these intervention-based DNA methylation alternations had minimal overlap with the differences seen in the healthy control samples.

**Conclusions:** Intervention-based cessation of alcohol use is associated with longitudinal site-specific DNA methylation differences.

**References**

1. Shenker NS, Polidoro S, Van Veldhoven K, Sacerdote C, Ricceri F, Birrell MA, Belvisi MG, Vineis P, Flanagan JM. Epigenome-wide association study in the European prospective investigation into cancer and nutrition (EPIC-Turin) identifies novel genetic loci associated with smoking. Hum Mol Genet. 2012;22:843–51.

2. Monick MM, Beach SR, Plume J, Sears R, Gerrard M, Brody GH, Philibert RA. Coordinated changes in AHRR methylation in lymphoblasts and pulmonary macrophages from smokers. Am J Med Genet B Neuropsychiatry Genet. 2012;159B(2):141–51.

3. Philibert RA, Plume JM, Gibbons FX, Brody GH, Beach SRH. The impact of recent alcohol use on genome wide DNA methylation signatures. Front Genet. 2012;3:54.

4. Philibert RA, Penaluna B, White T, Shires S, Gunter T, Liesveld J, Erwin C, Hollenbeck N, Osborn T. A pilot examination of the genome-wide DNA methylation signatures of subjects entering and exiting short-term alcohol dependence treatment programs. Epigenetics. 2014;9(9):1212–219.

## A10 Airway epithelial production of IL-17C in response to bacterial and rhinovirus co-exposure

### Kyla C. Jamieson, David Proud

#### Department of Physiology and Pharmacology, University of Calgary, Calgary, AB, Canada

##### **Correspondence:** Kyla C. Jamieson - kyla.jamieson@ucalgary.ca

*Allergy, Asthma & Clinical Immunology* 2016, **12(Suppl 2)**:A10

**Background:** Human rhinovirus (HRV) is the dominant viral pathogen associated with asthma exacerbations in both pediatric [1] and adult populations [2]. Bacterial pathogens also have been linked to wheezing episodes in children, with asymptomatic bacterial colonization at 1 month being linked to asthma development by age 5 [3]. In adult asthmatics, bacterial colonization has been associated with increased duration of asthma, and exacerbations within the past year [4]. Non-typeable *Haemophilus influenzae* (NTHI) is among the most common bacterial species detected in the airways of severe asthmatic patients [1, 2]. Importantly, bacterial and viral pathogens were commonly detected concurrently during wheezing episodes in children under 3 years old [1]. Further, asthmatic patients who smoke have more poorly controlled disease, and more frequent exacerbations than asthmatics who do not smoke [5]. Bacteria, viruses, and smoke primarily interact with the airway epithelium, but the mechanisms by which they promote asthma development or exacerbations are unclear. IL-17C is a novel cytokine reported to be increased in the bronchial epithelium during NTHI infection, and suggested to have pro-inflammatory and anti-bacterial functions in the airway. The effect of viral exposure on IL-17C has not been examined previously. We hypothesize human bronchial epithelial (HBE) cells initiate IL-17C production in response to respiratory pathogens as a protective anti-microbial mechanism.

**Methods:** Confluent primary normal HBE cells were treated with NTHI and/or HRV for up to 24 h. Other stimuli included replication-deficient HRV, the synthetic double-stranded RNA poly(I:C), and agonists of individual TLRs. Furthermore, HBE cells were exposed to medium or pathogens in the presence or absence of cigarette smoke extract (CSE). IL-17C mRNA and protein levels were measured using qRT-PCR and ELISA.

**Results:** Neither NTHI, nor HRV-16 alone induced significant IL-17C release. By contrast, co-exposure to NTHI and HRV-16 induced synergistic IL-17C protein release at 24 h, with mRNA upregulated as early as 6 h. This response was not HRV serotype-specific as it was observed with both HRV-16 and HRV-1A. Induction of IL-17C was dependent upon viral replication and could be mimicked by poly(I:C). Individual TLR agonists did not synergize with HRV to induce IL-17C. Finally, CSE inhibits the pathogen-induced IL-17C response.

**Conclusion:** Co-exposure to intact NTHI and replicating HRV stimulates synergistic IL-17C protein production in normal HBE cells, and is reduced by acute cigarette smoke exposure. Further studies are needed to clarify the potential role of IL-17C in respiratory infections in the airways of asthmatics and smokers with asthma.

**References**

1. Carlsson CJ, Vissing NH, Sevelsted A, et al. Duration of wheezy episodes in early childhood is independent of the microbial trigger. J Allergy Clin Immunol. 2015;136:1208–14.

2. Iikura M, Hojo M, Koketsu R, et al. The importance of bacterial and viral infections associated with adult asthma exacerbations in clinical practice. PLoS ONE. 2015;10:e0123584.

3. Bisgaard H, Hermansen MN, Buchvald F, et al. Childhood asthma after bacterial colonization of the airway in neonates. N Engl J Med. 2007;357:1487–95.

4. Zhang Q, Illing R, Hui CK, et al. Bacteria in sputum of stable severe asthma and increased airway wall thickness. Respir Res. 2012;13:35.

5. Althuis MD, Sexton M, Prybylski D. Cigarette smoking and asthma symptom severity among adult asthmatics. J Asthma. 1999;36:257–64.

## A11 Resiquimod (S28463) treatment prevents the increase in airway resistance and decreases inflammation and serum IgE levels in *Ascaris suum* induced allergic asthma model in non-human primates

### Cynthia Kanagaratham^1^, Pierre Camateros^2,3^, Frantisek Kopriva^4^, Jennifer Henri^5^, Marian Hajduch^4^, Danuta Radzioch^1,2^

#### ^1^Department of Human Genetics, McGill University, Montréal, QC, Canada, ^2^Division of Experimental Medicine, Faculty of Medicine, McGill University, Montréal, QC, Canada, ^3^Department of Medicine, Faculty of Medicine, University of British Columbia, Vancouver, BC, Canada, ^4^Faculty of Medicine and Dentistry, Institute of Molecular and Translational Medicine, Palacky University, Olomouc, Czech Republic, ^5^McGill University Health Center – Research Institute, Montréal, QC, Canada

##### **Correspondence:** Danuta Radzioch - danuta.radzioch@mcgill.ca

*Allergy, Asthma & Clinical Immunology* 2016, **12(Suppl 2)**:A11

**Background:** Allergic asthma is a disease affecting the respiratory and immune systems. Peptides targeting toll like receptors (TLRs) have been explored as drugs for treating allergic asthma. In published studies using acute allergic asthma model in C57Bl/6 and A/J mice and chronic asthma model in Brown Norway rats, we demonstrated the therapeutic potential of TLR 7/8 ligand, resiquimod S28463 (S28) [1, 2]. Here, we validated our findings from the rodent models in a non-human primate (NHP, *Macaca fascicularis*) model of allergic asthma.

**Methods:** We developed a protocol for establishing allergic asthma in non-allergic NHPs by sensitization and challenge to *Ascaris suum (A. suum)* antigen. NHPs were treated with S28 by nasogastric intubation 24 h prior to antigen challenge. The potential of S28 as a treatment for allergic asthma was assessed by measuring airway responsiveness to methacholine exposure, serum IgE concentration, and by quantifying the concentration of inflammatory cytokines and cells in bronchoalveolar lavage fluid (BALF).

**Results:** Sensitization followed by challenge with *A. suum* creates a successful allergic response in initially non-allergic NHPs. In allergic untreated animals, we observed an increase in serum IgE concentration, airway responsiveness, and inflammation in the BALF. Treatment with S28 prior to each allergen challenge caused a significant decrease in skin wheal area and total serum IgE concentration. S28 also prevented the increase in airway resistance observed with each subsequent allergen challenge. The level of inflammatory cells and cytokines in S28 treated and challenged animals were significantly different from challenged and untreated animals, and similar to those challenged with saline.

**Conclusion:** The data presented herein confirm that S28 has great potential as a therapeutic agent for allergic asthma. Studies on the molecular mechanisms responsible for this effect, as well as the potential when S28 is combined with other anti-asthma drugs remain to be explored.

**Acknowledgements:** This work was supported by funds from Sandler Program for Asthma Research (SPAR), the Canadian Institutes of Health Research (CIHR), Fonds de recherche du Québec-Santé (FRQS), and AllerGen NCE Inc., a member of the Networks of Centers of Excellence Canada program.

**References**

1. Moisan J, Camateros P, Thuraisingam T, Marion D, Koohsari H, Martin P, Boghdady ML, Ding A, Gaestel M, Guiot MC et al. TLR7 ligand prevents allergen-induced airway hyperresponsiveness and eosinophilia in allergic asthma by a MYD88-dependent and MK2-independent pathway. Am J Physiol Lung Cell Mol Physiol. 2006;290(5):L987–95.

2. Camateros P, Tamaoka M, Hassan M, Marino R, Moisan J, Marion D, Guiot MC, Martin JG, Radzioch D. Chronic asthma-induced airway remodeling is prevented by toll-like receptor-7/8 ligand S28463. Am J Respir Crit Care Med. 2007;175(12):1241–49.

## A12 Maternal depression during pregnancy and 4-month infant gut immunoglobulin A levels

### Liane J. Kang^1^, Petya T. Koleva^1^, Catherine J. Field^2^, Angela Chow^3^, Tedd Konya^4^, Malcolm R. Sears^5^, Padmaja Subbarao^6^, Piushkumar J. Mandhane^1^, Stuart E. Turvey^7^, Allan B. Becker^8^, James A. Scott^4^, Anita L. Kozyrskyj^1,9^

#### ^1^Department of Pediatrics, University of Alberta, Edmonton, AB, Canada, ^2^Department of Agricultural, Food and Nutritional Science, University of Alberta, Edmonton, AB, Canada, ^3^Department of Applied Health Science, Indiana University, Bloomington, IN, USA, ^4^Dalla Lana School of Public Health, University of Toronto, Toronto, ON, Canada, ^5^Department of Medicine, McMaster University, Hamilton, ON, Canada, ^6^Department of Pediatrics, University of Toronto, Toronto, ON, Canada, ^7^Department of Pediatrics, University of British Columbia, Vancouver, BC, Canada, ^8^Department of Pediatrics and Child Health, University of Manitoba, Winnipeg, MN, Canada, ^9^School of Public Health, University of Alberta, Edmonton, AB, Canada

##### **Correspondence:** Liane J. Kang - ljkang@ualberta.ca

*Allergy, Asthma & Clinical Immunology* 2016, **12(Suppl 2)**:A12

**Background:** Secretory immunoglobulin A (sIgA) has a critical role in early life gut mucosal immunity and is a marker of immune maturation. Delayed IgA production is associated with increased risk of allergic diseases [1]. Animal studies of stressful events before birth and during infancy show changes in the vaginal microbiome, as well as changes in intestinal microbial composition and lower sIgA concentrations in offspring [2, 3]. A first report in humans found infants born to mothers with greater stress during pregnancy more likely have gut dysbiosis [4], but there is a paucity of literature on stress-microbiome-immunity pathways in humans. This study investigated differences in total infant fecal IgA levels at 4 months according to the depression status of the mother before or during pregnancy.

**Methods:** The data were obtained from a sub-sample of 47 term infants from the Vancouver and Winnipeg sites of the Vanguard cohort of the Canadian Healthy Infant Longitudinal Development (CHILD) Study. Mothers of the infants were enrolled during pregnancy and were asked to report depression previously or currently through a standardized questionnaire. Pre/postnatal stress and depressive symptoms were ascertained from scored-scales administered to the general CHILD cohort for comparison. Infant stool samples were collected at a mean age of 3.9 months, and total fecal IgA was measured using an enzyme-linked immunosorbent assay. Mann–Whitney U-tests were used to detect differences in IgA levels using IBM SPSS version 23.

**Results:** Five trajectories for depressive symptoms and perceived stress in mothers (chronic, antepartum, postpartum, never-high scores, never-low scores) during pregnancy and at postpartum were found in the general cohort. About 8% of women had depressive symptoms during pregnancy. In the Vanguard cohort, 32% of mothers reported depression before or during pregnancy and 11% during pregnancy only. There was no statistically significant difference in fecal IgA between infants born from depressed and not depressed mothers (p = 0.63) in the Vanguard cohort; however, the median IgA in infants of mothers that were depressed is lower than in infants of not depressed mothers (10.9 (IQR = 4.5–27.8) vs. 13.5 (IQR = 7.2–36.4)ug/g of total protein).

**Conclusions:** Infants born to mothers with depression before or during pregnancy appear to have lower fecal IgA levels at 4 months. The small sample size and measurement of total IgA in place of sIgA may have prevented detection of statistical significance.

**Acknowledgements:** Support was provided by CIHR and AllerGen NCE. We acknowledge participants and investigators of the CHILD Study.

**References**

1. Kukkonen K, Kuitunen M, Haahtela T, Korpela R, Poussa T, Savilahti E. High intestinal IgA associates with reduced risk of IgE-associated allergic diseases. Pediatr Allergy Immunol. 2010;21:67–73.

2. Jasarevic E, Howerton CL, Howard CD, Bale TL. Alterations in the vaginal microbiome by maternal stress are associated with metabolic reprogramming of the offspring gut and brain. Endocrinology. 2015;156(9):3265–76.

3. Galley JD, Bailey MT. Impact of stressor exposure on the interplay between commensal microbiota and host inflammation. Gut Microbes. 2014;5(3):390–96.

4. Zijlmans MA, Korpela K, Riksen-Walraven JM, de Vos WM, de Weerth C. Maternal prenatal stress is associated with the infant intestinal microbiota. Psychoneuroendocrinology. 2015;53:233–45.

## A13 Linking the indoor microbiome with atopy in the CHILD Study: preliminary findings

### Theodore Konya^1^, Meghan B. Azad^6^, Jeff Brook^1,3^, Tim K. Takaro^4^, David Guttman^1^, Malcolm R. Sears^5^, Allan Becker^6^, Anita L. Kozyrskyj^2^, Padmaja Subbarao^1^, Piush Mandhane^2^, Stuart Turvey^7^, James Scott^1^, and CHILD Study Investigators^8^

#### ^1^University of Toronto, Toronto, ON, Canada, ^2^University of Alberta, Edmonton, AB, Canada, ^3^Environment Canada, Toronto, ON, Canada, ^4^Simon Fraser University, Burnaby, BC, Canada, ^5^McMaster University, Hamilton, ON, Canada, ^6^University of Manitoba, Winnipeg, MN, Canada, ^7^University of British Columbia, Vancouver, BC, Canada, ^8^Canadian Healthy Infant Longitudinal Development Study, Hamilton, ON, Canada

##### **Correspondence:** Theodore Konya - tedd.konya@utoronto.ca

*Allergy, Asthma & Clinical Immunology* 2016, **12(Suppl 2)**:A13

**Background:** Microbial communities of the indoor built environment have long been studied, largely using culture techniques; however, recent studies employing next-generation community sequencing analyses have suggested that indoor microbial communities are much more diverse and complex than previously thought [1]. How the make-up of the indoor microbiome affects the long-term health of the individuals within is not entirely clear.

**Methods:** Samples of household dust were obtained from 80 homes in Winnipeg, Canada, occupied by a selection of subjects enrolled in the Canadian Healthy Infant Longitudinal Development (CHILD) Study when the children were 3–4 months of age. Skin prick testing for sensitization to common food and environmental allergens and standardized physician’s diagnosis for atopic dermatitis was obtained for each child in the study at 1 and 3 years of age. For each dust sample, community bacterial partial 16S rDNA was sequenced using MiSeq (Illumina). Simpson’s diversity indices of variable groups were compared by Kruskal–Wallis test using False Discovery Rate to correct for multiple test comparisons.

**Results:** Dust samples were dominated by members of the phyla Actinobacteria, Bacteroidetes, Firmicutes, and Proteobacteria, with *Staphylococcus*, *Corynebacterium*, and *Streptococcus* as the highest-abundance genera. The bacterial diversity was generally higher in homes of children who were reported to have atopic dermatitis or were sensitized to food allergens on skin testing. Further, we observed significant differences (p < 0.05) in the bacterial diversity of the dust in homes of children with vs children without: atopy (1 year p = 0.018 and 3 years p = 0.038) and food sensitivity (1 year p = 0.018 and 3 years p = 0.004).

**Conclusion:** These results provide preliminary findings that reveal a link between the microbiome of the indoor environment and allergic health outcomes early in a child’s life. We recognize the N for the allergic groups is small. Therefore these results highlight the need for funding to further analyze the dust microbiome to increase power and provide a clearer snapshot of the entire CHILD cohort.

**Acknowledgements:** Funding for this project was provided by AllerGen NCE for the Better Exposure Avoidance Measures (BEAM) project. Thank you to the CHILD Study and Investigators for access to the dust samples and health outcomes data. Major funding for the CHILD Study provided by the Canadian Institutes for Health Research and AllerGen NCE.

**Reference**

1. Kelley ST and Gilbert JA. Studying the microbiology of the indoor environment. Genome Biol. 2013;14:202.

## A14 Maternal depression during pregnancy and fecal short chain fatty acid levels in infants

### Manjeet Kumari^1^, Angela Chow^2^, Sarah L. Bridgman^1^, Mon Tun^3^, Rupasri Mandal^4^, David S. Wishart^4^, Malcolm R. Sears^5^, Padmaja Subbarao^6^, Piushkumar J. Mandhane^1^, Stuart E. Turvey^7^, Allan B. Becker^8^, James A. Scott^9^, Anita L. Kozyrskyj^1,3^

#### ^1^Department of Pediatrics, University of Alberta, Edmonton, AB, Canada, ^2^Department of Applied Health Science, Indiana University, Bloomington, IN, USA, ^3^School of Public Health, University of Alberta, Edmonton, AB, Canada, ^4^The Metabolomics Innovation Centre, Edmonton, AB, Canada, ^5^Department of Medicine, McMaster University, Hamilton, ON, Canada, ^6^Department of Pediatrics, University of Toronto, Toronto, ON, Canada, ^7^Department of Pediatrics, University of British Columbia, Vancouver, BC Canada, ^8^Department of Pediatrics and Child Health, University of Manitoba, Winnipeg, MN, Canada, ^9^Dalla Lana School of Public Health, University of Toronto, Toronto, ON, Canada

##### **Correspondence:** Manjeet Kumari - mkumari@ualberta.ca

*Allergy, Asthma & Clinical Immunology* 2016, **12(Suppl 2)**:A14

**Background:** Maternal stress and depression can affect the composition of gut microbiota in infants. Gut microbiota produce large quantities of active metabolites, such as butyrate and other short-chain fatty acids (SCFA). Changes in microbial composition that alter metabolite levels have the capacity to influence host intestinal cells, including those of the immune system. SCFAs have recently been associated with anti-inflammatory activity not only in the gut, but also in peripheral tissue, such as the lungs [1]. We hypothesized an association between maternal depression and altered infant fecal SCFA levels which might induce allergic inflammation in offspring. This study investigated differences in infant fecal SCFA levels at 4 months according to the depression status of the mother during and after pregnancy.

**Methods:** This was a sub-study of 240 mother-infant dyads from the Edmonton, Vancouver and Winnipeg sites of the Canadian Healthy Infant Longitudinal Development (CHILD) birth cohort. The Center for Epidemiologic Studies Depression (CES-D) questionnaire was administered to women at recruitment (mean 27 weeks) and 36 weeks of gestation, and postnatally at 6 months, 1, 1.5 and 2 years. Based on CES-D scores, pre and postnatal trajectories for maternal depression were identified. Short chain fatty acid (SCFA) concentrations (acetate, butyrate, propionate, valerate) were measured by NMR as mmol/L in fecal samples obtained from infants at a mean age of 3.8 months. Statistically significant associations between maternal depression trajectory membership and infant fecal SCFA levels were tested by ANOVA using SPSS version 23.

**Results:** Five trajectories for maternal depressive symptoms during and after pregnancy (chronic, antepartum only, postpartum only, never-high scores and never-lower scores) were identified in the CHILD cohort. Women in the postpartum trajectory had comparable CES-D scores during pregnancy to women in the never depressed trajectory. Fecal butyrate levels in infants born to mothers in the antepartum depression trajectory were numerically higher (mean 1.71 mmol/L, N = 12, p < 0.06) than in infants of mothers in the postpartum depression trajectory (mean 0.77 mmol/L, N = 12), and of mothers with never-lower depression scores during and after pregnancy (mean = 1.05 mmol/L, N = 143, p < 0.07).

**Conclusions:** We report evidence of a tentative association between maternal depression during pregnancy and higher levels of butyrate produced by microbiota in fecal samples of infants at 4 months of age.

**Acknowledgements:** Support was provided by CIHR and AllerGen NCE. We acknowledge participants and investigators of the CHILD Study.

**Reference**

1. Thorburn AN, McKenzie C, Shen S, Stanley D, Macia L, Mason LJ et al. Evidence that asthma is a developmental origin disease influenced by maternal diet and bacterial metabolites. Nat Commun. 2015;6:7320.

## A15 Integration of transcriptomics, proteomics and genome-wide association studies (GWAS) with NetworkAnalyst to gain insights into innate immunity

### Amy H.Y. Lee^1,2^, Jeff Xia^3^, Erin Gill^1,2^, Bob Hancock^1,2,4^

#### ^1^Centre for Microbial Diseases and Immunity Research, University of British Columbia, Vancouver, BC, Canada, ^2^Department of Microbiology and Immunology, University of British Columbia, Vancouver, BC, Canada, ^3^Department of Parasitology, McGill University, Montréal, QC, Canada, ^4^Wellcome Trust Sanger Institute, Hinxton, UK

##### **Correspondence:** Amy H.Y. Lee - amy.lee@ubc.ca

*Allergy, Asthma & Clinical Immunology* 2016, **12(Suppl 2)**:A15

**Background:** Advances in high-throughput omics technologies allow us to quantify global cellular changes in DNA, RNA, proteins or metabolites under various experimental conditions or disease states. One active area of research is to develop methodology for integrating these large datasets to improve our mechanistic understanding and provide systems-level insights into cellular function. Furthermore, data integration can increase our statistical power as well as removing study bias. Here, we present NetworkAnalyst [1, 2], an interactive, web-based tool for biologists to perform complex meta-analysis, visual data mining and data integration.

**Methods:** There are two common approaches to data integration: (1) horizontal data integration, which combines different data sets from the same omic approach, such as performing meta-analysis of gene expression changes to identify expression signatures; and (2) vertical data integration, which seeks to coalesce different omic data sets, such as integrating transcriptomic and metabolomics data to identify perturbation in pathways of interest. To link these different datasets, NetworkAnalyst mines InnateDB [3] for curated protein–protein interactions (PPI) and creates biologically meaningful networks. Network topology analysis can be used to identify those protein nodes that are highly connected (i.e. hubs), which can serve as useful biomarkers or therapeutic targets; while densely connected units (i.e. modules) that show strong differential expression patterns can be used to identify unique disease signatures.

**Results:** Using NetworkAnalyst, we can generate novel hypotheses on various aspects of immune regulation, from neonatal vaccination response to common mis-regulation that contributes to inflammatory diseases such as asthma, Vasculitis and sepsis. For instance, we typically identify mis-regulated modules containing S100 protein family members in patients with inflammatory disorder. In patients who developed sepsis, we observed up-regulation in S100A8 and S100A9 genes and down-regulation in S100A4 gene [4]. A number of these S100 proteins have emerged as potential biomarkers in independent studies [5].

In contrast, we used NetworkAnalyst to overlay proteomic data onto transcriptomic networks to characterize neonatal response to vaccination. We observed the up-regulation of a key immune hub, STAT1, in our transcriptome analyses. Independently, we identified known STAT1 PPI in our proteomic analyses. Together, our data suggest STAT1 may play an important role in immunization response against viral vaccines.

**Conclusions:** Biological network analysis is a powerful approach to study complex diseases, and can be an invaluable tool for biomarker discovery and the identification of targets for therapeutic interventions.

**References**

1. Xia J, Benner MJ, Hancock RE. NetworkAnalyst: integrative approaches for protein–protein interaction network analysis and visual exploration. Nucleic Acid Res. 2014;42(W1):W167–74.

2. Xia J, Gill EE, Hancock RE. NetworkAnalyst for statistical, visual and network-based meta-analysis of gene expression data. Nat Protoc. 2015;10(6):823–44.

3. Lynn DJ, Windsor GL, Chan C, Richard N, Laird MR, Barsky A et al. InnateDB: facilitating systems-level analyses of the mammalian innate immune response. Mol Syst Biol. 2008; 4:218

4. Pena OM, Hancock DG, Lyle NH, Linder A, Russell JA, Xia J et al. An endotoxin tolerance signature predicts sepsis and organ dysfunction at initial clinical presentation. EBioMedicine. 2014;1(1):64–71

5. Donato R, Cannon BR, Sorci G, Riuzzi F, Hsu K, Weber DJ et al. Functions of S100 Proteins. Curr Mol Med. 2013;13(1):24–57

## A16 Novel approach for the identification of bronchial epithelial cells in lower human airways using flow cytometry

### Danay Maestre, Darren Sutherland, Jeremy Hirota, Olga Pena, Christopher Carlsten

#### Department of Medicine, University of British Columbia, Vancouver, BC, Canada

##### **Correspondence:** Danay Maestre - danay@mail.ubc.ca

*Allergy, Asthma & Clinical Immunology* 2016, **12(Suppl 2)**:A16

**Background:** The airway epithelial cell is the initial cell type impacted by inhaled environmental factors, such as pathogens, allergens, and pollutants [1]. Sampling lower airway body fluids like bronchoalveolar lavage (BAL), provides valuable information on the reaction of the lung to inhaled materials [2]. Conventional methods to discriminate bronchial epithelial cells (BEC) in lower airway samples include cytochemical staining, immunohistochemical procedures, standard and confocal microscopy and in situ hybridization [3]. These techniques present throughput limitations in the number of cells being quantified and time efficiency. Flow cytometry is an important tool that allows delineation of specific cell components of immune responses and disease states [4]. A well-defined flow cytometry panel for the identification of BEC in human lung fluids is currently lacking and we aimed to satisfy the existing need.

**Methods:** Human BAL and blood samples were obtained from volunteers enrolled in an ethics-approved clinical study at the Air Pollution Laboratory at The University of British Columbia. Blood samples, BEAS-2B (BEC Cell line) and primary BEC isolated from bronchial brushings were used as negative and positive control respectively. Collection and processing of BAL and blood samples was performed following standard operation procedures in order to lyse and remove red blood cells and clumped tissue. 

Cells were examined by side scatter area (SSC-A) versus forward scatter area (FSC-A), then using forward scatter height versus FSC-A to eliminate debris and clumped cells from the analysis. Single cells were sub-gated using Fixable Viability Dye eFluor^®^ 450 and subsequently live cells were discriminated by the expression of CD45 surface marker conjugated to the flourochrome Allophycocyanin/Cy7 (APC-Cy7). Exclusion of CD45 positive cells, commonly used marker for total leukocytes, was followed by the examination of double expression of Fluorescein Isothiocyanate conjugated Pan-Cytokeratin (PanCK-FITC) intracellular marker versus PerCP-Cyanine5.5 conjugated EpCAM (EpCAM PerCP-Cy5.5).

**Results:** Bronchial epithelial cells collected from human BAL samples were detected by the proposed panel combining intracellular and surface markers. The percentages found in BAL (4.6%), positive controls (92 ± 5%) and blood samples (0.0%) were consistent with the literature when other non-flow cytometric methods were used.

**Conclusions:** We developed a protocol for the accurate identification and quantification of BEC using flow cytometry.

**Acknowledgements:** This work was supported by the Vancouver Coastal Health Research Institute and AllerGen NCE. We thank Mandy Pui and SzeWing Wong for generous efforts on earlier stages of this project, and Min Ryu and Christopher Rider for kindly providing the BEC primary cells and cell line.

**References**

1. Proud D, Leigh R. Epithelial cells and airway diseases. Immunol Rev. 2011;242(1):186–204.

2. Henderson RF. Use of bronchoalveolar lavage to detect respiratory tract toxicity of inhaled material. Exp Toxicol Pathol. 2005; 57(SUPPL. 1):155–59.

3. Finotto S, Rado V, Dal Vecchio A, Milani G, Fabbri M, Maestrelli P. Identification of epithelial cells in bronchoalveolar lavage. Hum Exp Toxicol. 1993;12(1):43–6.

4. Yu YRA, Hotten DF, Malakhau Y, Volker E, Ghio AJ, Noble PW et al. Flow cytometric analysis of myeloid cells in human blood, bronchoalveolar lavage, and lung tissues. Am J Respir Cell Mol Biol. 2016;54(1):13–24. doi:10.1165/rcmb.2015-0146OC.

## A17 DNA methylation profiles unique to a longevity region: Nicoya, Costa Rica

### Lisa M. McEwen^1^, Meaghan J. Jones^1^, Julia L. MacIsaac^1^, William H. Dow^2,3^, Luis Rosero-Bixby^2,3^, Michael S. Kobor^1^, David H. Rehkopf^4^

#### ^1^Department of Medical Genetics, University of British Columbia, Vancouver, BC, Canada, ^2^Centro Centroamericano de Población, Universidad de Costa Rica, San José, Costa Rica, ^3^Department of Demography, University of California, Berkeley, CA, USA, ^4^Division of General Medical Disciplines, School of Medicine, Stanford University, Stanford, CA, USA

##### **Correspondence:** Lisa M. McEwen - lmcewen@cmmt.ubc.ca

*Allergy, Asthma & Clinical Immunology* 2016, **12(Suppl 2)**:A17

**Background:** Aging is an inevitable biological process, involving several molecular and physiological changes, which exhibit substantial heterogeneity across individuals. The Nicoya Peninsula in Costa Rica represents a population having one of the highest demographically validated old age life expectancies in the world [1]. The increased lifespan has been hypothesized as a result of environmental exposures and lifestyle factors such as nutrition, exercise, and social status; however, the reasons for this longevity remain uncharacterized from the perspective of underlying biological mechanisms. DNA methylation is a process that is responsive to environmental influences and has the potential to regulate gene expression, with particular trends that are highly correlated to chronological age [2]. Given the different environmental exposures these populations experience, DNA methylation is most likely acting as a possible mediator between Nicoyan individuals and the longevity observed.

**Methods:** We assessed DNA methylation of whole blood in 95 Costa Ricans, aged between 60 and 110 years old, using the Illumina 450k Methylation Array. Blood cell proportions were estimated using DNA methylation using a well established reference based method [3].

**Results:** We identified a significant difference in the DNA methylation-based predicted proportion of two age-associated cell types: CD8+ naïve and CD8+ memory T cells. Interestingly, CD8+ naïve T cells decrease with age, whereas CD8T+ memory cells increase. The proportion of CD8+ naïve cells was higher and CD8+ memory T cells was lower in Nicoyan samples, respectively, when compared to control samples from surrounding non-longevity areas of Costa Rica (CD8T+ naïve: p value <0.01, CD8T + memory: p value <0.05).

**Conclusions:** Results may represent a product of environmental influence that may explain this suggested immunologically younger phenotype in Nicoya. In summary, by DNA methylation profiling we were able to detect a biological distinction of cellular abundance that may potentially be representative of a slow-aging marker in an ethnically novel and unique centenarian cohort.

**References**

1. Rehkopf DH, Dow WH, Rosero-Bixby L, Lin J, Epel ES, Blackburn EH. Longer leukocyte telomere length in Costa Rica’s Nicoya Peninsula: a population-based study. Exp Gerontol. 2013;48(11):1266–73.

2. Horvath S. DNA methylation age of human tissues and cell types. Genome Biol. 2013;14(10):R115.

3. Houseman E, Accomando WP, Koestler DC, Christensen BC, Marsit CJ, Nelson HH, et al. DNA methylation arrays as surrogate measures of cell mixture distribution. BMC Bioinform. 2012;13(1):86.

## A18 Evaluating the function of bronchial epithelial cells and their associated cytokine expressions of IL-17A and IL-17F

### Takeshi Morimoto, Steven G. Smith, John-Paul Oliveria, Suzanne Beaudin, Abbey Schlatman, Karen Howie, Caitlin Obminski, Graeme Nusca, Roma Sehmi, Gail M. Gauvreau, Paul M. O’Byrne

#### Department Medicine, McMaster University, Hamilton, ON, Canada

##### **Correspondence:** Takeshi Morimoto - takeshi@mcmaster.ca

*Allergy, Asthma & Clinical Immunology* 2016, **12(Suppl 2)**:A18

**Background:** Th17 cell produce IL-17, which is an important cytokine in inflammatory airway disease. Recent reports indicate that other cells are capable of producing IL-17 including B cells, Neutrophils NKT-cells [1]. IL-17 has six subtypes (from IL-17A to IL-17F), but there are structural similarities between IL-17A and IL-17F. Particularly, IL-17A and IL-17F bind to the same receptor subunits, IL-17 receptor A (IL-17RA) and IL-17RC heterodimer [2]. However, IL-17A and IL-17F have different roles in inflammation. IL-17A is associated with allergic inflammation, while IL-17F is associated mainly with infectious inflammation mainly. IL-17A and IL-17F are known to stimulate bronchial epithelial cells (BEC) to produce cytokines like IL-6, CXCL-1 and GM-CSF. In our study, we evaluated and compared the surface expression of IL-17RA and intracellular expressions of IL-17A, IL-17F, IL-6 and CXCL-1 in BEC between asthma, COPD and healthy subjects. In addition, we analyzed the relationship between these receptors and cytokines.

**Methods:** We collected sputum cells from mild asthmatics (n = 10), subjects of mild to moderate COPD (n = 8), and healthy control (n = 8). All asthmatics and COPD subjects had stable airway disease and were not on inhaled or oral corticosteroid therapy. We stained sputum cells with fluorescent labeled antibodies and relevant isotype controls, and evaluated the expressions of IL-17RA, IL-17A, IL-17F, IL-6 and CXCL-1 in BEC and CD4+ lymphocytes using flow cytometry (Becton–Dickinson LSRII). We used expression of CD45− and CD326+ to detect BEC [3]. Data was analyzed using FlowJo software.

**Results:** There were no significant differences in the expression of IL-17RA, IL-17A, IL-17F, IL-6 and CXCL-1 in BEC between asthmatic, COPD and healthy subjects. However, there was a positive correlation between surface expression of IL-17RA and intracellular expression of IL-17A, IL-17F and IL-6 in BEC in all subjects. In addition, we found a positive correlation between intracellular expression of IL-17A in CD4+ lymphocytes and surface expression of IL-17RA in BEC.

**Conclusions:** The current study did not find significant differences in the expression of cytokines and receptors associated with IL-17A and IL-17F in BEC. However, positive correlations between (1) IL-17A in CD4+ lymphocytes and IL-17RA in BEC and (2) surface expression of IL-17RA and intracellular expressions of IL-17A, IL-17F and IL-6 in BEC suggest that IL-17A production by CD4+ lymphocytes may drive BEC production of IL-17A and IL-17F, through IL-17RA. Further investigation is required to fully understand the mechanistic relationship between CD4+ lymphocyte derived cytokines and activation of BEC.

**References**

1. Chesné J, Braza F, Mahay G, Brouard S, Aronica M, Magnan A. IL-17 in severe asthma. Where do we stand? Am J Respir Crit Care Med. 2014;190(10):1094–1101.

2. Gaffen SL. Structure and signaling in the IL-17 receptor family. Nat Rev Immunol. 2009;9(8):556–67.

3. Fujino N, Kubo H, Ota C, Suzuki T, Suzuki S, Yamada M et al. A novel method for isolating individual cellular components from the adult human distal lung. Am J Respir Cell Mol Biol. 2012;46(4):422–30.

## A19 Prenatal smoke exposure alters mitochondrial DNA methylation in umbilical cord blood dendritic cells

### Michelle North^1^, Cheng Peng^2^, Marco Sanchez-Guerra^2^, Hyang-Min Byun^3^, Jeff Brook^1,4^, Anne K. Ellis^5^, Andrea A. Baccarelli^2^

#### ^1^Southern Ontario Centre for Atmospheric Aerosol Research, University of Toronto, ON, Canada, ^2^Laboratory of Environmental Epigenetics, Department of Environmental Health, Harvard T.H. Chan School of Public Health, Boston, MA, USA, ^3^Human Nutrition Research Centre, Institute of Cellular Medicine, Newcastle University, Newcastle upon Tyne, UK, ^4^Air Quality Research Division, Environment Canada, Toronto, ON, Canada, ^5^Department of Medicine, Queen’s University, Allergy Research Unit, Kingston General Hospital, ON, Canada

##### **Correspondence:** Anne K. Ellis - ellisa@kgh.kari.net; Andrea A. Baccarelli - abaccare@hsph.harvard.edu

*Allergy, Asthma & Clinical Immunology* 2016, **12(Suppl 2)**:A19

**Background:** Epigenetic alterations, including changes in DNA methylation, are related to various perinatal environmental factors that affect child health, such as maternal smoking, C-section, gestational weight gain and maternal allergy [1–7]. Human cells contain nuclear and mitochondrial DNA (mtDNA), but the impact of mitochondrial epigenetics on the development of environmentally-linked diseases, such as allergies and asthma, has not been adequately explored. Mitochondria are particularly sensitive to oxidative stress, responding with changes in copy number and mtDNA methylation [8, 9]. Dendritic cells were targeted because of their sensitivity to environmental stimuli and importance in allergic disease [10–13]. We tested the hypothesis that prenatal smoke exposure, a source of oxidative stress during fetal development, would be associated with mitochondrial epigenetic differences in dendritic cells at birth.

**Methods:** Umbilical cord blood dendritic cells were isolated using magnetic sorting (n = 91). Samples were drawn from a cohort with a known high prevalence of maternal smoking, the Kingston Allergy Birth Cohort [14]. We analyzed mtDNA regions with potential functional impacts, including the D-loop promoter, transfer RNA phenylalanine (MTTF), and 12S ribosomal RNA (MT-RNR1) by pyrosequencing [8]. Copy number was determined using qPCR. Copy number and mtDNA were analyzed for associations with perinatal factors using models adjusted for maternal age, pre-pregnancy BMI, ethnicity, child’s gender, and SES.

**Results:** The subset of the cohort with umbilical cord blood available for mtDNA analyses did not exhibit significant differences from the Kingston Allergy Birth Cohort as a whole in terms of maternal age, pre-pregnancy BMI, weight gain during pregnancy, sex of the child, mode of delivery, siblings, maternal allergy, income, gestational age or maternal exposure to cigarette smoke. Prenatal smoke exposure was associated with a 1.53% (95% CI 0.61–2.46%, p = 0.002, adjusted model) increase in mtDNA methylation in MTTF. The D-loop mtDNA region in dendritic cell mitochondria also demonstrated an increase in methylation associated with maternal smoke exposure during pregnancy, of 3.82% (95% CI 0.50–7.14%, p = 0.03, adjusted model). However, we did not observe significant associations between mtDNA methylation and gestational weight gain, maternal allergy or C-section delivery. MtDNA copy number was also not associated with any of the perinatal risk factors examined.

**Conclusions:** Maternal smoking was associated with differences in umbilical cord blood dendritic cell mitochondrial DNA methylation in 2 out of the 3 regions examined. These effects on mtDNA may be related to the known effects of smoking on oxidative stress balance, and may affect dendritic cell function and the development of allergic disease.

**References**

1. Hou L, Zhang X, Wang D, Baccarelli A. Environmental chemical exposures and human epigenetics. Int J Epidemiol. 2012;41:79–105.

2. Sofer T, Baccarelli A, Cantone L, Coull B, Maity A, Lin X, Schwartz J. Exposure to airborne particulate matter is associated with methylation pattern in the asthma pathway. Epigenomics. 2013;5:147–54.

3. Burke H, Leonardi-Bee J, Hashim A, Pine-Abata H, Chen Y, Cook DG, Britton JR, McKeever TM. Prenatal and passive smoke exposure and incidence of asthma and wheeze: systematic review and meta-analysis. Pediatrics. 2012;129:735–44.

4. Azad MB, Konya T, Guttman DS, Field CJ, Sears MR, HayGlass KT, et al. Infant gut microbiota and food sensitization: associations in the first year of life. Clin Exp Allergy. 2015;45:632–43.

5. Camargo CA. Gestational weight gain and offspring asthma: a novel opportunity for primary prevention research Clin Exp Allergy 2015;45:544–6.

6. Miller RL, Ho SM. Environmental epigenetics and asthma: current concepts and call for studies. Am J Respir Crit Care Med. 2008;177:567–73.

7. Prescott SL. Early-life environmental determinants of allergic diseases and the wider pandemic of inflammatory noncommunicable diseases. J Allergy Clin Immunol. 2013;131:23–30.

8. Byun HM, Panni T, Motta V, Hou L, Nordio F, Apostoli P, Bertazzi PA, Baccarelli AA, Effects of airborne pollutants on mitochondrial DNA methylation. Part Fibre Toxicol. 2013;10:18.

9. Byun HM, Baccarelli AA. Environmental exposure and mitochondrial epigenetics: study design and analytical challenges. Hum Genet. 2014;133:247–57.

10. Fedulov AV, Kobzik L. Allergy risk is mediated by dendritic cells with congenital epigenetic changes. Am J Respir Cell Mol Biol. 2011;44:285–92.

11. Gaurav R, Agrawal DK. Clinical view on the importance of dendritic cells in asthma. Expert Rev Clin Immunol. 2013;9:899–919.

12. Kuo CH, Hsieh CC, Kuo HF, Huang MY, Yang SN, Chen LC, Huang SK, Hung CH, Phthalates suppress type I interferon in human plasmacytoid dendritic cells via epigenetic regulation. Allergy. 2013;68:870–9.

13. Porter M, Karp M, Killedar S, Bauer SM, Guo J, Williams D, Breysse P, Georas SN, Williams MA. Diesel-enriched particulate matter functionally activates human dendritic cells. Am J Respir Cell Mol Biol. 2007;37:706–19.

14. Perinatal Health Report 2008. BORN Ontario 2010; Eastern Ontario Public Health Region.

## A20 Polydimethylsiloxane as versatile passive air sampler for measuring levels of phthalates indoors

### Joseph O. Okeme^1^, Suman Dhal^2^, Aman Saini^1^, Miriam L. Diamond^1,2,3^

#### ^1^Department of Physical and Environmental Science, University of Toronto Scarborough, Toronto, ON, Canada, ^2^Dalla Lana School of Public Health, University of Toronto, Toronto, ON, Canada, ^3^Department of Earth Sciences, University of Toronto, Toronto, ON, Canada

##### **Correspondence:** Joseph O. Okeme - joe.okeme@mail.utoronto.ca

*Allergy, Asthma & Clinical Immunology* 2016, **12(Suppl 2)**:A20

**Background:** Phthalates are plasticizers used in a variety of consumer building products including vinyl flooring and wallpapers, and personal care products such as cosmetics. Exposure to phthalates that are released from these sources have been linked to increased risk of asthma, allergies and other auto-immune diseases [1]. A step towards limiting human exposure to phthalates is to measure their abundance. Active air samplers are conventionally used for this purpose but their use is hindered by cost, need for electrical power, skilled operation and their noise [2]. Passive air samplers (PAS), which work by diffusion, are efficient alternatives to active samplers. They are cheap, noise-free, have no power and minimal skill requirements [2]. We have developed a PAS from commonly available rubber, polydimethylsiloxane (PDMS). PDMS collects and retains a wide range of chemicals and is easy to use [3]. Owing to its versatility, PDMS can be used for stationary sampling of micro-environments, monitoring human personal exposure and even exposure of wildlife. This project was aimed at developing a versatile and easily used passive air sampler.

**Methods:** PDMS stationary samplers were deployed in 22 Toronto homes for one month in the summer of 2013. In a personal exposure study, two participants wore PDMS broaches in their breathing zone while working at their desks for 8 h daily for a total of 4 days, in the fall of 2015. Post-deployment samples were extracted and analysed for phthalates using gas chromatography mass spectrometry (GC–MS).

**Results:** Average concentration of ∑_6_ phthalates ranged from 1153 ± 421 to 1351 ± 556 ng/m^3^ in bedrooms and living rooms, respectively. No significant difference (p > 0.01) was found between bedroom and living room levels, indicating the ubiquitous and well-mixed nature of phthalates and indoor air, respectively. PDMS worn as a personal passive air sampler was able to detect phthalates after 1 day.

**Conclusion:** This study shows the feasibility and versatility of using PDMS as a passive air sampler for measuring levels of phthalates in the indoor environment.

**Acknowledgements:** We thank AllerGen NCE and the Natural Science and Engineering Research Council (NSERC) for funding this research.

**References**

1. Bertelsen RJ, Carlsen KCL, Calafat AM, Hoppin JA, Håland G, Mowinckel P, Carlsen KH, Løvik M. Urinary biomarkers for phthalates associated with asthma in Norwegian children. EHP. 2013;121(21):251–6.

2. Shoeib M, Harner T. Characterization and comparison of three passive air samplers for persistent organic pollutants. Env Sci Tech. 2002;36(19):4142–51.

3. Okeme JO, Saini A, Yang C, Zhu J, Smedes F, Klànovà J, Diamond ML. Calibration of polydimethylsiloxane and XAD-Pocket passive air samplers (PAS) for measuring gas- and particle-phase SVOCs indoors. Atm Env. 015.

## A21 Effect of inhaled allergen challenge on eosinophil and neutrophil activation in subjects with mild allergic asthma following inhaled allergen challenge

### Christopher J. Olesovsky^1^, John-Paul Oliveria^1^, Brittany M. Salter^1^, Michael Wang^1^, Suzanne Beaudin^1^, Paige Lacy^2^, Roma Sehmi^1^, Gail M. Gauvreau^1^

#### ^1^Department Medicine, McMaster University, Hamilton, ON, Canada, ^2^Department Medicine, University of Alberta, Edmonton, AB, Canada

##### **Correspondence:** Christopher J. Olesovsky - chrisolesovsky@live.ca

*Allergy, Asthma & Clinical Immunology* 2016, **12(Suppl 2)**:A21

**Background:** Eosinophils and neutrophils migrate to the site of allergic stimulation and contribute to airway hyper-responsiveness and airway remodelling through the release of toxic granule proteins, reactive oxygen species, cytokines and lipid mediators [1, 2]. Our understanding of systemic activation of these cells following allergic stimulation of the airways is limited. This study evaluated the expression of activation markers on circulating eosinophils and neutrophils, and levels of their degranulation products, eosinophil peroxidase (EPX) and neutrophil elastase (NE), collected before and after whole lung allergen inhalation challenges (AIC) in mild allergic asthmatics.

**Methods:** Eleven subjects with mild allergic asthma were challenged with inhaled allergen and developed early and late phase asthmatic responses. Blood and sputum samples were collected at baseline, 7 and 24 h following AIC. Blood cells were stained with antibodies to identify neutrophil (CD45+ , CD16+) and eosinophil (CD45+ , CD16−) populations, and activation was determined by expression of CD11b, CD18, CD35, CD62L, CD63, and CD66b [3, 4]. Sputum samples were processed using dithiothreitol (DTT). EPX and NE levels in sputum supernatant and plasma samples were measured by ELISA. GraphPad Prism was used to perform 1-way ANOVAs and Tukey post hoc analyses; data are presented as mean ± SEM.

**Results:** At 7 h post-challenge there was a significant increase in eosinophil expression of CD18 (1.6-fold), CD62L (1.8-fold) CD63 (1.6-fold), and a significant increase in neutrophil expression of CD35 (2.27-fold) (p < 0.05). At 24 h post-challenge there was a significant increase in eosinophil expression of CD18 (1.9-fold), CD35 (1.2-fold), CD62L (2.3-fold), and a significant increase in neutrophil expression of CD18 (2.04-fold) and CD63 (1.83-fold) (p < 0.05). Plasma levels of EPX were significantly lower at 7 h (134.6 ± 5.10 ng/ml) and 24 h (129.9±3.30 ng/ml) post AIC compared to baseline (150.1 ± 5.09 ng/ml; p < 0.05). There were no statistically significant changes in plasma and sputum supernatant levels of NE and sputum supernatant levels of EPX at 7 and 24 h following AIC compared to baseline.

**Conclusions:** We demonstrate that circulating eosinophils and neutrophils respond similarly in their upregulation of some markers of activation following a whole lung AIC. This shared response may be beneficial when developing therapies targeting granulocytes for treatment of asthma. In contrast to other reports, EPX levels decreased slightly in the circulation post-challenge, and this decrease may suggest lung-homing of these cells [5]. No increase of sputum EPX or NE levels following AIC contrasts previous reports, and may reflect the method of sample processing and poor stability of proteins during storage.

**References**

1. Possa SS, Leick EA, Prado CM, Martins MA, Tibério IFLC. Eosinophilic inflammation in allergic asthma. Front Pharmacol. 2013;4:46

2. Monteseirín J. Neutrophils and asthma. J Investig Allergol Clin Immunol. 2009;19:340–54.

3. Pelikan Z. Expression of surface markers on the blood cells during the delayed asthmatic response to allergen challenge. Allergy Rhino. 2014;5:96–109.

4. Mahmudi-Azer S. Translocation of the tetraspanin CD63 in association with human eosinophil mediator release. Blood. 2002;99:4039–47.

5. Erpenbeck VJ et al. Local release of eosinophil peroxidase following segmental allergen provocation in asthma. Clin Exp Allergy. 2003;33:331–6.

## A22 Airway epithelial cells reduce airway smooth muscle cell contractility

### Michael J. O’Sullivan^1^, Chan Y. Park^2^, Jeffrey J. Fredberg^2^, Anne-Marie Lauzon^1^, James G. Martin^1^

#### ^1^Department of Physiology, McGill University, Montréal, QC, Canada, ^2^Department of Environmental Health, Harvard School of Public Health, Boston, MA, 02115, USA

##### **Correspondence:** James G. Martin - james.martin@mcgill.ca

*Allergy, Asthma & Clinical Immunology* 2016, **12(Suppl 2)**:A22

**Background**: Airway smooth muscle (ASM) plays a critical role in modulating airway diameter. ASM cells are considered to exist in one of two phenotypes at any given time; contractile or proliferative [1]. Co-culture of ASM with airway epithelial cells has been reported to induce proliferation but effects on the contractile properties are unexplored [2]. The aim of the current study was to examine whether ASM cells cultured in the presence of epithelial cells have a reduced contractile phenotype.

**Methods**: Primary ASM and normal human bronchial epithelial cells (NHBE) were harvested from control subjects. Epithelial cell line, BEAS-2B, was also utilized. Epithelial cells cultured on Transwell^®^ permeable supports were placed in culture with ASM for 24 h before calcium or force responses to histamine were measured by fura-2 imaging or traction force microscopy respectively. Protein and RNA was collected for analysis by Western blot and qPCR.

**Results**: ASM co-cultured with epithelial cells demonstrated a reduction in force generation and calcium release after stimulation with 1 μM histamine. Co-cultured cells had reduced mRNA and protein of specific contractile apparatus proteins. We observed no change in the expression of caveolin-1, or the expression of mRNA governing proteins responsible for calcium release, including CD38, IP_3_R, PLCβ or SERCA. COX-2 and mPGES-1 mRNA were increased after co-culture, suggesting a possible phenotype switch towards a pro-inflammatory state. Pre-treatment with the COX-1 inhibitor indomethacin and SC-560 restored calcium signalling after histamine stimulation.

**Conclusions**: Airway epithelial cells cause a reduction in ASMC contractility in vitro that is not transcriptionally regulated. Further examination of COX-2 products may provide explanations for this observed phenotype.

**Acknowledgements:** Supported by a grant from the CRRN of CTS and FRSQ.

**References**

1. Halayko AJ, Solway J. Molecular mechanisms of phenotypic plasticity in smooth muscle cells. J Appl Physiol. 1985. 2001;90(1):358–68.

2. Malavia NK et al. Airway epithelium stimulates smooth muscle proliferation. Am J Respir Cell Mol Biol. 2009;41(3):297–304.

## A23 Diesel exhaust and allergen co-exposure enhances mucin secretome in human airways

### Min Hyung Ryu^1,3^, Neeloffer Mookherjee^2,3^, Christopher Carlsten^1,3^

#### ^1^Department of Respiratory Medicine, University of British Columbia, Vancouver, BC, Canada, ^2^Department of Internal Medicine and Immunology, University of Manitoba, Winnipeg, MN, Canada, ^3^Canadian Respiratory Research Network (CRRN), Ottawa, ON, Canada

##### **Correspondence:** Min Hyung Ryu - ryum@mail.ubc.ca

*Allergy, Asthma & Clinical Immunology* 2016, **12(Suppl 2)**:A23

**Background:** Diesel exhaust (DE) is the most significant source of urban ultrafine traffic-related air pollution. In a controlled human exposure study, DE exposure has been demonstrated to augment allergen-induced airway inflammation [1]. However, tissue specific changes driving the synergy from allergen-DE co-exposure remains poorly elucidated.

**Methods:** We performed a randomized, double-blinded, controlled human crossover study. Five mild asthmatics, sensitized to the study allergen, inhaled filtered air or DE (300 mg/m^3^) for 2 h. Subsequently, one lung segment was challenged with allergen, and saline was administered to a contralateral segment. Bronchoalveolar lavage (BAL) and endobronchial biopsies were obtained 48 h after allergen challenge. This was repeated 4 weeks later, but for opposite inhalation and new segments for the allergen challenge. Four experimental conditions were derived: FAS (filtered air and saline), DES (diesel exhaust and saline), FAA (filtered air and allergen) and DEA (diesel exhaust and allergen). BAL from five subjects were pooled for each condition and processed by LC–MS/MS using a label-free quantitative proteomics approach. Endobronchial biopsies from five subjects were immunostained for different mucins (MUC), such as Muc5AC, Muc4 and Muc16.

**Results:** Proteins were selected with >99% confidence criteria (i.e. at least two non-redundant peptides identified with a log(e) ≤3). The resulting expression matrix contained more than 2500 proteins across all four conditions. Expression intensity was determined by spectral counting. Relative fold changes in FAA, DES and DEA were calculated compared to FAS. Co-exposure with DEA significantly altered the expression of 76 proteins compared to FAA and 130 proteins compared to DES. These studies showed that Muc5AC, Muc4 and Muc16 were elevated more than eightfold with DEA co-exposure compared to either allergen or DE exposures alone. Muc5B was detected at similar levels with DE alone and DEA co-exposure. None of the mucins detected were enhanced by allergen alone, whereas Muc2 and Muc7 were detected only in the DEA secretome and not in any of the other three conditions.

**Conclusions:** This is the first study to demonstrate that DE and allergen co-exposure enhances specific glycoproteins in the lungs, which may be associated with increased induction of mucus production. Changes in the mucus composition following DE-allergen co-exposure may contribute to the synergistic effect of DE exposure on allergen-induced airway inflammation.

**Acknowledgements:** This work was supported by funding from the BC Lung Association and AllerGen-NCE. We acknowledge the technical expertise of Peyman Ezzati and Victor Spicer for the proteomics studies.

**Reference**

1. Carlsten C, Blomberg A, Pui M, Sandstorm T, Wong SW, Alexis N, Hirota J. Diesel exhaust augments allergen-induced lower airway inflammation in allergic individuals: a controlled human exposure study. Thora. 2015;0:1–10.

## A24 Sensitization and allergy to highly-allergenic foods at age 3 years

### Elinor Simons^1^, Diana Lefebvre^2^, David Dai^2^, Allan B. Becker^1^, Stuart Turvey^3^, Padmaja Subbarao^4^, Piushkumar Mandhane^5^, Malcolm Sears^2^

#### ^1^Section of Allergy and Immunology, Department of Pediatrics and Child Health, University of Manitoba, Winnipeg, MN, Canada, ^2^Division of Respirology, Department of Medicine, McMaster University, Hamilton, ON, Canada, ^3^Division of Allergy and Immunology, Department of Pediatrics, British Columbia Children’s Hospital, Vancouver, BC, Canada, ^4^Department of Pediatrics, Hospital for Sick Children, Toronto, ON, Canada, ^5^Division of Pediatric Respirology, Pulmonary & Asthma, University of Alberta, Edmonton, AB, Canada

##### **Correspondence:** Elinor Simons - elinor.simons@umanitoba.ca

*Allergy, Asthma & Clinical Immunology* 2016, **12(Suppl 2)**:A24

**Background:** We evaluated the associations between the development of sensitization and food allergy diagnoses for milk, egg and peanut at age 3 years, with the goal of contributing to our understanding of why only some food-sensitized children develop an allergy to the food.

**Methods:** Caregivers of participants in the population-based Canadian Healthy Infant Longitudinal Development (CHILD) Study prospectively reported their child’s introduction and reactions to foods in the first 3 years of life. At ages 1 and 3 years, the children underwent skin prick testing to foods including milk, egg and peanut and assessment by an Allergist or trained researcher for food allergies and other allergic conditions. We evaluated the patterns of food sensitization and healthcare provider-diagnosed food allergies at age 3 years.

**Results:** In preliminary data (n = 2644), sensitization (skin prick test mean wheal diameter ≥2 mm) was 1.10% to cow milk, 2.31% to egg and 3.87% to peanut at age 3 years. Between ages 1 (n = 2935) and 3 years, some children lost sensitization to milk (1.40%), egg (5.58%) and peanut (2.35%) and others were newly sensitized (0.74, 0.78 and 1.37%, respectively). Diagnoses of allergy to milk, egg and peanut were made for 13.8, 16.4 and 21.6% of children sensitized to each of these foods, respectively. Of children with a diagnosis of allergy to milk, egg and peanut, 40.0, 58.8 and 84.6% were sensitized to the food to which they were allergic. Sensitized children had higher odds of being diagnosed with an allergy (70-fold increase for milk and egg, and 170-fold increase for peanut).

**Conclusion**: Most children with sensitization to milk, egg and peanut at age 3 years were not diagnosed with an allergy to these foods; however, sensitization was a strong predictor of allergy diagnosis. Future work will examine dietary and other predictors of food sensitization and allergy to further our understanding of how sensitization to a food becomes a food allergy and ultimately, to work towards the prevention of food allergy development.

**Acknowledgements:** We acknowledge the CHILD Study Investigators for their contributions. The CHILD study is supported by AllerGen NCE and the Canadian Institute of Health Research. This research is supported by the Children’s Hospital Research Institute of Manitoba and the University of Manitoba.

## A25 Novel blood-based biomarker panels of the late phase asthmatic response: from discovery to validation

### Amrit Singh^1,2^, Casey P. Shannon^2^, Young Woong Kim^1,2^, Chen Xi Yang^1,2^, Gail M. Gauvreau^3^, J. Mark FitzGerald^1^, Louis-Philippe Boulet^4^, Paul M O’Byrne^3^, Scott J. Tebbutt^1,2^

#### ^1^Department of Medicine, University of British Columbia, Vancouver, BC, Canada, ^2^Prevention of Organ Failure (PROOF) Centre of Excellence, Vancouver, BC, Canada, ^3^Department of Medicine, McMaster University, Hamilton, ON, Canada, ^4^Centre de Pneumologie de L’Hôpital, Université Laval, Québec City, QC, Canada

##### **Correspondence:** Amrit Singh - amrit.singh@hli.ubc.ca

*Allergy, Asthma & Clinical Immunology* 2016, **12(Suppl 2)**:A25

**Background:** We have previously shown significant molecular changes in the blood between asthmatic individuals who elicit the dual response (dual responders, DRs) compared to those that develop the isolated early response (early responders, ERs) after allergen inhalation challenge [1, 2]. Identifying individuals likely to develop the dual response may aid in the screening of subjects for clinical trials which test drugs for the attenuation of the late phase asthmatic response or provide novel targets for therapeutics. The objective of this study was to develop blood-based biomarker panels that could identify asthmatic individuals with high probability of developing a late phase response.

**Methods:** The discovery cohort consisted of 36 asthmatic individuals (15 ERs and 21 DRs) and an independent cohort of 45 (9 ERs and 36 DRs) asthmatic individuals made up the validation cohort. Blood samples were collected prior to allergen challenge. After RNA extraction, and globin depletion the total RNA was sequenced using an Illumina HiSeq 2000 as 100 bp paired end reads at Genome Quebec. Both genome-guided datasets such as UCSC genes, UCSC gene-isoforms, and Ensembl and de novo assembled transcripts using the Trinity software [3] were constructed. Top ranked biomarker candidates were transferred to the clinically relevant nanoString platform and final biomarker panels were identified and locked down and validated in the external cohort.

**Results:** Predictive biomarker panels had a classification performance (based on the area under the receiver operating curve, AUC) that ranged between 60 and 70% in the discovery cohort. 87 transcripts identified on the RNA-Seq platform were transferred to the nanoString elements platform. The transcripts were split with respect to their dataset of origin and validated in the external cohort. The UCSC gene-isoforms and Trinity biomarker panels had an AUC of 67 and 71%, respectively. The UCSC gene-isoforms panel was enriched with genes from the TCR Signaling pathway whereas the Trinity panel was enriched with Signaling by the B Cell Receptor (BCR) pathway.

**Conclusions:** Predictive gene biomarker panels in the blood are successful at identifying individuals likely to develop the late response upon allergen inhalation challenge. These biomarker panels consisted of novel transcripts that are representative of T cell and B cell biology. These panels will be evaluated in other allergic conditions such as allergic rhinitis where a similar phenomenon of the biphasic response exists.

**References**

1. Singh A, Yamamoto M, Kam SHY, Ruan J, Gauvreau GM, O’Byrne PM, FitzGerald JM, Schellenberg R, Boulet LP, Wojewodka G, Kanagaratham C, De Sanctis JB, Radzioch D, Tebbutt SJ. Gene-metabolite expression in blood can discriminate allergen-induced isolated early from dual asthmatic responses. PLoS ONE. 2013;8:e67907.

2. Singh A, Yamamoto M, Ruan J, Choi JY, Gauvreau GM, Olek S, Hoffmueller U, Carlsten C, FitzGerald JM, Boulet LP, others. Th17/Treg ratio derived using DNA methylation analysis is associated with the late phase asthmatic response. Allergy Asthma Clin Immunol. 2014;10:32.

3. Haas BJ, Papanicolaou A, Yassour M, Grabherr M, Blood PD, Bowden J, Couger MB, Eccles D, Li B, Lieber M, MacManes MD, Ott M, Orvis J, Pochet N, Strozzi F, Weeks N, Westerman R, William T, Dewey CN, Henschel R, LeDuc RD, Friedman N, Regev A. De novo transcript sequence reconstruction from RNA-seq using the Trinity platform for reference generation and analysis. Nat Protoc. 2013;8:1494–1512.

## A26 Characterization of IgE receptor expression in human airway epithelial cells

### Gurpreet K. Singhera, S. JasemineYang, Delbert R. Dorscheid

#### Centre for Heart Lung Innovation, University of British Columbia, Vancouver, BC, Canada

##### **Correspondence:** Gurpreet K. Singhera - gurpreet.singhera@hli.ubc.ca

*Allergy, Asthma & Clinical Immunology* 2016, **12(Suppl 2)**:A26

**Background:** The human airway epithelium serves as the first line of defense against environmental exposures to maintain immune homeostasis. Asthma is an airways inflammatory disease characterized by epithelial barrier dysfunction and cytokine imbalance. Immunoglobulin E (IgE) is known to play a key role in initiating inflammatory responses in asthma. IgE has two receptors, the high affinity receptor, FcεRI and low affinity receptor, FcεRII (CD23). FcεRI is a multi-chain receptor comprising of four polypeptide chains: one alpha (α), one beta (β), two gamma (γ) units. FcεRII (CD23) has two isoforms-CD23a and Cd23b. Expression and distribution of FcεRI and its isomers can contribute to initiating inflammatory responses. The expression of IgE receptors is not well characterized in the airway epithelium. In this study we are examining the expression of IgE receptors in the airway epithelium and investigating their role in mediating allergic inflammation.

**Methods:** In an in vitro model, monolayer cultures of primary human airway epithelial cells (AEC) from non-asthmatic and asthmatic donors were treated with human and mouse IgE. Total RNA and protein lysates were collected for qPCR and western blot analysis for IgE receptor expression. Cell-free conditioned media were collected to quantify pro-inflammatory mediators by ELISA. IgE-binding to FcεRI was confirmed using a cell-based assay. IgE-induced FcεRI activation was detected by phosphorylation of downstream kinases.

**Results:** Primary human AEC isolated from asthmatic and non-asthmatic lungs demonstrated significantly increased FcεRIα protein compared to non-asthmatic cells. mRNA expression of FcεRI β subunit and FcεRII (CD23b) subunits was significantly increased in asthmatic AEC. IgE binding to FcεRI on AEC was demonstrated in a cell based assay post-treatment with varying doses of IgE. IgE binding was inhibited in a dose dependent manner when AEC were pre-treated with the anti-FcεRI antibody that specifically targeted the IgE binding site. Activation of kinases downstream of FcεRI was observed in response to IgE treatment in primary non-asthmatic AEC.

**Conclusion:** Understanding of IgE receptor expression and function in airway epithelial cells will help us to further investigate the mechanism of allergic inflammation.

## A27 Determinants and variability of docosahexaenoic acid (DHA) content in human milk in the CHILD Study: implications for allergic disease

### Hasantha Sinnock^1^, Susan Goruk^2^, Catherine J. Field^2^, Allan B. Becker^1^, Piushkumar J. Mandhane^2^, Padmaja Subbarao^3^, Stuart E. Turvey^4^, Malcolm R. Sears^5^, Meghan B. Azad^1^

#### ^1^Department of Pediatrics and Child Health, University of Manitoba and Children’s Hospital Research Institute of Manitoba, Winnipeg, MN, Canada, ^2^Department of Pediatrics, University of Alberta, Edmonton, AB, Canada, ^3^Department of Pediatrics, Hospital for Sick Children, University of Toronto, Toronto, ON, Canada, ^4^Department of Pediatrics, Child & Family Research Institute and BC Children’s Hospital, University of British Columbia, Vancouver, BC, Canada, ^5^Department of Medicine, McMaster University, Hamilton, ON, Canada

##### **Correspondence:** Hasantha Sinnock - hasantha.sinnock@umanitoba.ca

*Allergy, Asthma & Clinical Immunology* 2016, **12(Suppl 2)**:A27

**Background:** An adequate supply of dietary Docosahexaenoic Acid (DHA) (C22:6, omega-3 fatty acid) is critical for human nutrition. Low DHA intake during infancy has been associated with an increased risk of chronic diseases including asthma and allergies [1, 2], and the DHA content in breastmilk varies widely between mothers [3, 4]. We aimed to characterize this variation according to maternal diet and other demographic, environmental and health factors in a national cohort of Canadian women.

**Methods:** We studied a representative sub-group of 400 mothers participating in the Canadian Healthy Infant Longitudinal Development (CHILD) Study from four sites across Canada (Vancouver, Edmonton, Manitoba and Toronto). A food frequency questionnaire administered during pregnancy was used to document maternal diet and estimate DHA intake. The DHA content of breastmilk samples collected at 3 months postpartum was determined by gas chromatography and expressed as percentage total fatty acids (%TFA). Linear regression models were used to determine associations between DHA content, estimated DHA intake and other maternal characteristics.

**Results:** The median DHA content in breastmilk among all mothers was 0.12 %TFA (interquartile range: 0.09– 0.19%) and the median estimated dietary DHA intake was 95 mg per day (interquartile range: 41–170 mg), originating mainly from fish and fortified dairy or egg consumption. DHA in breastmilk was strongly correlated with dietary DHA intake (r = +0.30, p < 0.001). Use of daily maternal multivitamins or fish oil supplements also predicted higher DHA in breastmilk (both p < 0.001). Independent of dietary DHA and supplement use, DHA in breastmilk was significantly lower among obese mothers, and significantly higher among mothers with a postsecondary degree, those of Asian ethnicity, and those residing in Vancouver. In a multivariate model adjusting for all of these dietary and non-dietary factors simultaneously, all were significant independent predictors of breastmilk DHA. Maternal age, asthma, allergy, and season of milk collection were not associated with DHA content in breastmilk.

**Conclusions:** Our results confirm that maternal diet and supplement use are key determinants of DHA content in breastmilk, and further identify several non-dietary factors that may influence the intake of this important bioactive nutrient among breastfed infants in Canada. These findings may be relevant to nutrition-based allergic disease prevention strategies. Ongoing research in the CHILD Study will extend analyses to evaluate additional fatty acids in breastmilk and explore associations with child health outcomes throughout early childhood.

**Acknowledgements:** This work was supported by AllerGen NCE Inc. (the Allergy, Genes and Environment Network), the Canadian Institutes of Health Research, the Manitoba Medical Services Foundation, and the Manitoba Children’s Hospital Research Foundation.

**References**

1. Miyata J, Arita M. Role of omega-3 fatty acids and their metabolites in asthma and allergic diseases. Allergol Int. 2015;64(1):27–34.

2. Tai EK, Wang XB, Chen ZY. An update on adding docosahexaenoic acid (DHA) and arachidonic acid (AA) to baby formula. Food Funct. 2013;4(12):1767–75.

3. Yuhas R, Pramuk K, Lien EL. Human milk fatty acid composition from nine countries varies most in DHA. Lipids. 2006;41(9):851–58.

4. Brenna JT, Varamini B, Jensen RG, Diersen-Schade DA, Boettcher JA, Arterburn LM. Docosahexaenoic and arachidonic acid concentrations in human breast milk worldwide. Am J Clin Nutr. 2007;85(6):1457–64.

## A28 Predictors of inappropriate usage of rescue medications in asthma: a 12-year population-based study

### Hamid Tavakoli^1,2^, J. Mark FitzGerald^1,2^, Larry D. Lynd^3^, Mohsen Sadatsafavi^1,2^

#### ^1^Institute for Heart and Lung Health, Department of Medicine, University of British Columbia, Vancouver, BC, Canada, ^2^Centre for Clinical Epidemiology and Evaluation, University of British Columbia, Vancouver, BC, Canada, ^3^Faculty of Pharmaceutical Sciences, University of British Columbia, Vancouver, BC, Canada

##### **Correspondence:** Hamid Tavakoli - hamid.tavakoli@ubc.ca

*Allergy, Asthma & Clinical Immunology* 2016, **12(Suppl 2)**:A28

**Background:** Despite strong evidence of harm, patients continue to receive excessive rescue medications such as short acting beta agonist agents (SABA). Understanding factors associated with the inappropriate use of SABA (IUoS) can help develop better policies to tackle this problem.

**Methods:** We used British Columbian (BC) administrative health data of between 2002 and 2013. We created a retrospective cohort of individuals between 14 and 55 years of age based on a validated asthma definition. The follow-up time was divided into adjacent 12-months periods. The outcome of interest was IUoS, as defined in a previously published algorithm [1]. Exposures were demographic variables at baseline and indicators of types and quality of care (fee code for spirometry, care provider specialty) as well as appropriate use of inhaled corticosteroid (ICS) in the prior year [2]. A generalized linear model was used to examine exposure-outcome associations, controlling for several potential confounding variables.

**Results:** 352,936 individuals (56% female, average age 30.2 at entry) satisfied the case definition, generating 2.6 million patient-years. On average, 18.5 % of patient-years were categorized as IUoS. The factor most strongly associated with IUoS was appropriate use of corticosteroid; (OR = 0.30, 95% CI 0.29–0.30). Female sex (OR = 0.95, 95% CI 0.94–0.97) and increasing age was associated with lower odds of inappropriate use, and increasing age OR = 0.96, 95% CI 0.96–0.97) was associated with a lower likelihood of inappropriate use Among quality of care indicators, use of spirometry was associated with 5% reduction in the odds of IUoS. Patients with a history of a visit to specialists including respiratory medicine, internal medicine, and allergy/immunization in the previous year showed 29, 22 and 14% lower likelihood of IUoS, respectively (Table 3).

**Table 3:** Results of the regression analysis of inappropriate use of SABA

Variable group	Variable	Odds ratio	95% CI (lower, upper)	p value
Patient characteristics	Sex (female = 1)	0.95	0.94–0.97	<.0001
	Socio economic score	0.98	0.97–0.98	<.0001
	Year	0.95	0.95–0.95	<.0001
	Age (per 10 years increase)	0.96	0.96–0.97	<.0001
Type and quality of care (Asthma attributable)	Pulmonary function test	0.95	0.93–0.98	<.0001
	Ratio of ICS to total medication more than 50%	0.30	0.29–0.30	<.0001
	Respirologist consultation	0.71	0.68–0.74	<.0001
	Cardiologist consultation	1.73	0.88–3.39	0.110
	Internal medicine consultation	0.78	0.75–0.81	<.0001
	Allergist consultation	0.86	0.82–0.89	<.0001
	General Practitioner visits			
	No visit	0.56	0.55–0.56	<.0001
	1 visit PY (Reference group)	–	–	–
	2 visits PY	0.77	0.76–0.78	<.0001
	More than 2 visits	0.53	0.52–0.54	<.0001
	Continuity of care (COC)			
	COC = 0 (Reference group)	–	–	–
	COC >0 and COC <50%	0.84	0.82–0.85	<.0001
	COC ≥50% and COC <100%	0.83	0.82–0.85	<.0001
	COC = 100%	0.85	0.83–0.87	<.0001
Comorbidity (Asthma attributable events are excluded)	Number of hospitalizations	1.01	1.00–1.02	0.143
	Number of physician visits	0.88	0.87–0.89	<.0001
	Modified Charlson score	1.00	0.99–1.01	0.130

**Conclusions:** Despite proven safety issues, inappropriate use of SABA still occurs among a significant minority of asthma patients, necessitating further research into its determinants. Several factors related to patient and process/quality of care are associated with inappropriate use of SABAs. Guidelines have not been successful in ensuring appropriate asthma treatment especially among patients under the care of primary care providers.

**Acknowledgements:** This study was funded through an arm’s length research contract with AstraZeneca Canada.

**References**

1. Anis AH, Lynd LD, Wang XH, King G, Spinelli JJ, FitzGerald M, Bai T, Paré P. Double trouble: impact of inappropriate use of asthma medication on the use of health care resources. CMAJ Can Med Assoc J J Assoc Medicale Can. 2001;164:625–631.

2. Laforest L, Licaj I, Devouassoux G, Chatte G, Martin J, Van Ganse E. Asthma drug ratios and exacerbations: claims data from universal health coverage systems. Eur Respir J. 2014;43:1378–86.

## A29 Comparative outcomes of the Nasal Allergen Challenge model of the Allergic Rhinitis Clinical Investigator Collaborative versus the Environmental Exposure Unit

### Mark W. Tenn^1^, Jenny Thiele^1,2^, Daniel E. Adams^1,2^, Lisa M. Steacy^1,2^, Anne K. Ellis^1,2^

#### ^1^Department of Biomedical and Molecular Sciences, Queen’s University, Kingston, ON, Canada, ^2^Allergy Research Unit, Kingston General Hospital, Kingston, ON, Canada

##### **Correspondence:** Mark W. Tenn - 0mwt2@queensu.ca

*Allergy, Asthma & Clinical Immunology* 2016, **12(Suppl 2)**:A29

**Background**: The Allergic Rhinitis Clinical Investigator Collaborative uses a Nasal Allergen Challenge (NAC) model to evaluate the clinical efficacy of novel therapeutics for allergic rhinitis (AR) and to study the cellular mechanisms and pathobiology of AR. Previous pilot studies have optimized the qualifying criteria and allergen concentration used in the NAC model. In the current study, we aimed to compare subjective nasal symptoms and objective nasal airflow measurements obtained with the NAC model to results from an Environmental Exposure Unit (EEU) study.

**Methods**: 11 birch allergic individuals who had participated in a study validating the use of birch pollen in the EEU were screened, with six completing the study. During screening, fourfold increases in birch allergen concentration from 1:128 were delivered intranasally every 15 min until a fall in peak nasal inspiratory flow (PNIF) of ≥50% from baseline and a total nasal symptom score (TNSS) ≥8/12 were obtained. The cumulative allergen concentration of all doses received at screening was used as the concentration to be delivered during the NAC visit. Three non-allergic participants were also enrolled. During the NAC visit, participants recorded their PNIF and TNSS at baseline (before challenge) and 15, 30 min, 1 h, and hourly until 12 h post-challenge. Nasal symptom scores from the EEU study were obtained for each participant and GraphPad Prism was used for the statistical analysis.

**Results**: An immediate sharp increase in TNSS was observed following NAC, with participants reaching peak TNSS 15 min post-challenge (p < 0.01). By contrast, in the EEU study, a gradual increase in TNSS was observed, with participants reaching peak TNSS after 3.5 h of continuous controlled birch pollen exposure (p < 0.0001). The % PNIF reduction mirrored these observations, with participants reaching peak % PNIF reduction at 30 min (p < 0.05) and 4 h (p < 0.05) during the NAC study and EEU exposures, respectively. Both the NAC and EEU were able to evoke a significant increase in TNSS (p = 0.0003 for NAC, p < 0.0001 for EEU) compared to baseline. A moderate (R^2^ = 0.58, p = 0.02) and strong (R^2^ = 0.83, p = 0.0006) correlation between TNSS and % PNIF reduction was observed for the NAC and EEU respectively.

**Conclusions**: Even though both the NAC and EEU are able to evoke nasal AR symptoms, the kinetics of this AR response differ between the two models. Industry partners should consider these differences when choosing an optimal allergen challenge model to use for the evaluation of their novel AR therapeutic agent.

## A30 Maintenance milk oral immunotherapy at 9 months is associated with ongoing increases in casein-specific serum IgG4

### Bahar Torabi^1^, Sarah De Schryver^1^, Duncan Lejtenyi^1^, Ingrid Baerg^2^, Edmond S. Chan^2^, Moshe Ben-Shoshan^1^, Bruce D. Mazer^1^

#### ^1^The Research Institute of the McGill University Health Centre, Montréal, QC, Canada, ^2^Division of Allergy & Immunology, Department of Pediatrics, BC Children’s Hospital, University of British Columbia, Vancouver, BC, Canada

##### **Correspondence:** Bahar Torabi - bahar.torabi@mail.mcgill.ca

*Allergy, Asthma & Clinical Immunology* 2016, **12(Suppl 2)**:A30

**Background:** Cow’s milk allergy (CMA) affects 2–5% of children [1] and is a cause of severe allergic reactions and anaphylaxis in children. Desensitization through milk oral immunotherapy (OIT) is an expanding area of research, where the duration of OIT for induction of tolerance remains unclear [2]. We report on the first randomized controlled trial in Canada evaluating milk oral immunotherapy (OIT) in children. We compared changes in skin prick tests (SPT) between commercial milk extract, fresh milk, and 1:10 diluted fresh milk (fresh milk-1:10) during milk OIT. We also assessed casein- specific serum IgE, IgG4, and IgA in subjects who successfully completed milk OIT.

**Methods:** We performed an interim analysis for the first 10 subjects who completed the escalation phase to 200 ml and for the first 5 subjects who continued maintenance doses for 9 months post escalation phase (9 M Post OIT). SPT to milk extract, fresh milk-1:10, and fresh milk were evaluated at baseline, 200 ml, and 9 M Post OIT. Casein- specific serum IgE, IgG4, and IgA were evaluated at baseline, 200 ml, and 9 M Post OIT. Statistical analysis was done using the paired *t* test.

**Results:** There was a statistically significant decrease in SPT from baseline to 200 ml for milk extract (mean difference 4.36 mm, 95% CI 0.78–7.95 mm), fresh milk-1:10 (mean difference 5.55 mm, 95% CI 1.62–9.47 mm), and fresh milk (mean difference 6.46 mm, 95% CI 2.13–7.05 mm**).** SPT did not significantly decrease at 9 M Post OIT compared to 200 ml. In parallel, casein-specific serum IgE decreased significantly from baseline to 200 ml (mean difference 14.41 ng/ml, 95% CI 1.70–21.11 ng/ml) and did not significantly decrease at 9 M Post OIT compared to 200 ml. Similarly, casein-specific IgA decreased significantly from baseline to 200 ml (mean difference 24.98 ng/ml, 95% CI 0.71–49.26 ng/ml), and did not significantly decrease at 9 M Post OIT compared to 200 ml. Serum IgG4 to casein increased significantly from baseline to 200 ml (mean difference 1255 μg/ml, 95% CI 28.75–2482 μg/ml), from baseline to 9 M Post OIT (mean difference 2582 μg/ml, 95% CI 614.99–4550 μg/ml), and from 200 ml to 9 M Post OIT (mean difference 1840 μg/ml, 95% CI 233.02–3446 μg/ml).

**Conclusion:** Successful escalation phase of milk OIT in IgE-mediated CMA in children is associated with decreases in milk-specific SPT and casein-specific serum IgE and IgA, and increases in casein-specific serum IgG4. There are further increases in IgG4 during maintenance milk OIT at 9 months post escalation phase. This suggests there are ongoing immunological changes during maintenance milk OIT and should be continued long-term.

**Acknowledgements:** This work was supported by AllerGen NCE (The Allergy, Genes and Environment Network), CIHR (Canadian Institutes of Health Research) and the Richard and Edith Strauss Clinical Fellowship.

**References**

1. Rona RJ, Keil T, Summers C, Gislason D, Zuidmeer L, Sodergren E, et al. The prevalence of food allergy: a meta-analysis. J Allergy Clin Immunol. 2007;120:638–46.

2. Yee CS, Rashid R. The heterogeneity of oral immunotherapy clinical trials: implications and future directions. Curr Allergy Asthma Rep. 2016;16(4):25.

## A31 The effects of infant feeding practices on food sensitization in a Canadian birth cohort

### Maxwell M. Tran^1^, Diana L. Lefebvre^1^, Wei Hao Dai^1^, Padmaja Subbarao^2^, Wendy Lou^3^, Allan B. Becker^4^, Piush J. Mandhane^5^, Stuart E. Turvey^6^, Malcolm R. Sears^1^

#### ^1^Department of Medicine, McMaster University, Hamilton, ON, Canada, ^2^Department of Paediatrics, University of Toronto, Toronto, ON, Canada, ^3^Dalla Lana School of Public Health, University of Toronto, Toronto, ON, Canada, ^4^Department of Pediatrics and Child Health, University of Manitoba, Winnipeg, MN, Canada, ^5^Department of Pediatrics, University of Alberta, Edmonton, AB, Canada, ^6^Department of Pediatrics, University of British Columbia, Vancouver, BC, Canada

##### **Correspondence:** Maxwell M. Tran - tranmm@mcmaster.ca

*Allergy, Asthma & Clinical Immunology* 2016, **12(Suppl 2)**:A31

**Background**: Evidence regarding the impact of infant feeding practices, including breastfeeding and timing and diversity of food introduction, on atopic sensitization remains controversial. We examined the relationship between infant feeding and development of sensitization to foods at age 1 in the Canadian Healthy Infant Longitudinal Development (CHILD) birth cohort study.

**Methods**: Nutrition questionnaire data prospectively collected at age 3, 6, 12, 18 and 24 months were used to characterize timing of introduction of cow’s milk products (CMP), egg, and peanut/peanut butter; diversity of food introduction; and exclusive breastfeeding to 6 months. At 1 year, infants underwent skin prick testing to cow’s milk, egg white, and peanut, with a wheal diameter ≥2 mm regarded as positive. To allow the ability to account for potential confounders, we analyzed data from children with complete data for timing of food introduction and skin prick testing for the infant and both parents (n = 1421).

**Results:** Most parents introduced CMP early (0–6 months 48%, 7–12 months 48%, ≥12 months 4%) but delayed introduction of egg (0–6 months 6%, 7–12 months 76%, ≥12 months 19%) and particularly peanut (0–6 months 1%, 7–12 months 41%, ≥12 months 58%). Exclusive breastfeeding to 6 months was low in our cohort (23%). At age 1 year, 10% of children were food-sensitized, with highest prevalence of egg white sensitization (6%). Early introduction of CMP, egg, and peanut was protective against sensitization to the corresponding food allergens. Introducing egg before age 1 significantly reduced the odds of developing sensitization to any of the three tested food allergens (0–6 months adjOR 0.33, 95% CI 0.12–0.90; 7–12 months adj OR 0.47, 95% CI 0.31–0.73), after adjusting for study center, parental atopy, and parental ethnicity. The benefits of early introduction of these foods in reducing the risk of sensitization were more evident in male children. Exclusive breastfeeding to 6 months did not affect the risk of sensitization to foods, except for cow’s milk (adj OR 4.66, 95% CI 1.31–16.61). Within the three allergenic foods assessed, less diversity of introduction before age 1 was associated with greater risk of food sensitization.

**Conclusions**: Early introduction of solid foods reduced the risk of food sensitization, especially in male children, as did an increased diversity of these allergenic foods introduced during the first year. The findings from this study reaffirm the paradigm shift from delayed food introduction and food avoidance to earlier introduction of diverse foods for allergy prevention.

**Acknowledgements:** The authors are grateful to the families who participated in this study, the CHILD Study team, and AllerGen NCE Inc. (the Allergy, Genes and Environment Network), a member of the Networks of Centres of Excellence Canada program.

## A32 Postnatal exposure to household cleaning products shape the infants’ gut microbiota composition at 3–4 months

### Mon H. Tun^1^, Petya T. Koleva^2^, Theodore Konya^3^, David S. Guttman^4^, Radha S. Chari^5^, Malcolm R. Sears^6^, Padmaja Subbarao^7^, Piushkumar J. Mandhane^2^, Stuart E. Turvey^8^, Allan B. Becker^9^, James A. Scott^3^, Jeffrey R. Brook^3^, Tim K. Takaro^10^, Anita L. Kozyrskyj^1,2^; and CHILD Study Investigators

#### ^1^School of Public Health, University of Alberta, Edmonton, AB, Canada, ^2^Department of Pediatrics, University of Alberta, Edmonton, AB, Canada, ^3^Dalla Lana School of Public Health, University of Toronto, Toronto, ON, Canada, ^4^Cell & Systems Biology, University of Toronto, Toronto, ON, Canada, ^5^Department Obstetrics and Gynecology, University of Alberta, Edmonton, AB, Canada, ^6^Department of Medicine, McMaster University, Hamilton, ON, Canada, ^7^Department of Pediatrics, University of Toronto, Toronto, ON, Canada, ^8^Department of Pediatrics, University of British Columbia, Vancouver, BC, Canada, ^9^Department of Pediatrics and Child Health, University of Manitoba, Winnipeg, MN, Canada, ^10^Faculty of Health Sciences, Simon Fraser University, Burnaby, BC, Canada

##### **Correspondence:** Mon H. Tun - tun@ualberta.ca

*Allergy, Asthma & Clinical Immunology* 2016, **12(Suppl 2)**:A32

**Background:** According to Health Canada, the average Canadian family uses 20–40 l of cleaning products each year [1]. In developed countries there is accumulating evidence of an increased risk for asthma and atopic disease with exposure to household cleaning products [2–5]. Infant gut microbiota dysbiosis has been linked to asthma and allergic disease. Hence, the aim of this study was to assess if the gut microbial composition of infants is affected by postnatal exposure to household cleaning agents.

**Methods:** The study involved 787 infants enrolled in the Canadian Healthy Infant Longitudinal Development (CHILD) birth cohort from Edmonton, Winnipeg and Vancouver sites. At 3 months after delivery, mothers were asked to complete questionnaires on aspects of their health, environment, lifestyle and personal use of household cleaning products namely: disinfectant (multi-surface cleaner), detergent and other chemicals (spray air-freshener). Fecal samples were collected at 3–4 months and fecal microbiota were characterized by Illumina high-throughput sequencing of the hyper-variable V4 region of the 16S rRNA gene. Bacterial taxon abundance, microbiota richness and diversity were compared between infants with high indoor disinfectant exposure (n = 414, 52.6%) and low indoor disinfectant exposure (n = 373, 47.4%) based on above and below median of total frequency scores, using Mann–Whitney U-test. Comparisons were corrected for multiple testing with the False Discovery Rate and stratified to adjust for confounding factors, such as infant antibiotic treatment.

**Results:** More than half of the CHILD cohort households used disinfectants at least once a month, mostly multi-surface cleaner. At 3–4 months of infant age, high indoor disinfectant exposure was associated with low fecal abundance of Actinobacteria (p = 0.0002) at the phylum level and of Bifidobacteriaceae (p = 0.021) at the family level. A similar trend was detected in a comparison restricted to vaginally delivered, exclusively breastfed infants not treated with antibiotics (directly and indirectly). Moreover, total microbial diversity was reduced at the order level (p = 0.028) in infants in the high exposure group. Additionally, *Pseudomonas* was more abundant in infants exposed to low disinfectant and high other chemicals compared to low disinfectant and low other chemicals (p = 0.012).

**Conclusion:** Our findings find evidence of an association between household disinfectant exposure and changes to infant gut microbial composition at 3–4 months of age. They suggest the possibility that cleaning products may affect development of the infant gut microbiome and immunity, with possible consequences for allergic disease later in life.

**Acknowledgements:** Sincere thanks to the CHILD research team and all study families. This research is supported by CIHR & AllerGen.

**References**

1. Evaluation Reports—Health Canada. Hc-sc.gc.ca. N.p. Web; 2016.

2. Bédard A, Varraso R, Sanchez M, Clavel-Chapelon F, Zock J, Kauffmann F, Le Moual N. Cleaning sprays, household help and asthma among elderly women. Respir Med. 2014;108:171–80.

3. Kimber I, Pieters R. Household chemicals, immune function, and allergy: a commentary. J Immunotoxicol. 2012;10:169–72.

4. Choi H, Schmidbauer N, Spengler J, Bornehag C. Sources of propylene glycol and glycol ethers in air at home. Int J Environ Res Public Health. 2010;7:4213–37.

5. Choi H, Schmidbauer N, Sundell J, Hasselgren M, Spengler J, Bornehag C. Correction: common household chemicals and the allergy risks in pre-school age children. PLoS ONE. 2011;6.

## A33 Impact of the hospital microbial environment on infant’s gut microbial composition at 3–4 months

### Mon H. Tun^1^, Petya T. Koleva^2^, Theodore Konya^3^, David S. Guttman^4^, Radha S. Chari^5^, Malcolm R. Sears^6^, Padmaja Subbarao^7^, Piushkumar J. Mandhane^2^, Stuart E. Turvey^8^, Allan B. Becker^9^, James A. Scott^3^, Anita L. Kozyrskyj^1,2^; and CHILD Study Investigators

#### ^1^School of Public Health, University of Alberta, Edmonton, Canada, ^2^Department of Pediatrics, University of Alberta, Edmonton, Canada, ^3^Dalla Lana School of Public Health, University of Toronto, Toronto, Canada, ^4^Cell & Systems Biology, University of Toronto, Toronto, Canada, ^5^Dept Obstetrics & Gynecology, University of Alberta, Edmonton, Canada, ^6^Department of Medicine, McMaster University, Hamilton, Canada, ^7^Department of Pediatrics, University of Toronto, Toronto, Canada, ^8^Department of Pediatrics, University of British Columbia, Vancouver, Canada, ^9^Department of Pediatrics and Child Health, University of Manitoba, Winnipeg, Canada

##### **Correspondence:** Mon H. Tun - tun@ualberta.ca

*Allergy, Asthma & Clinical Immunology* 2016, **12(Suppl 2)**:A33

**Background:** Hospital acquired infections (HAIs) affect 8% of Canadian children, with higher prevalence rates found in infants 12% [1]. In Canada, the average length of hospital stay after vaginal delivery is about 2 days but post caesarean delivery it is 4 days [2]. An extended hospital stay increases infant exposure to the hospital microbial environment and elevates the risk for gut colonization with opportunistic microorganisms at a time when the seeding of intestinal microbiota in infants is critical in shaping their immune system. Asthma and atopic disease has been associated with gut microbiota dysbiosis during infancy.

**Objective:** To determine the impact of infant exposure to the hospital environment on the composition of gut microbiota.

**Methods:** A subset of 787 fecal samples from the Canadian Healthy Infant Longitudinal Development (CHILD) birth cohort from Edmonton, Winnipeg and Vancouver sites were included in this study. Data on infant hospital length-of-stay at birth and afterwards was obtained from birth chart reviews and maternal report at 3 months postpartum. Infant gut microbiota at 3–4 months was characterized by Illumina 16S rRNA sequencing. Microbial relative abundance, Shannon diversity and Chao1 species richness were determined. The gut microbial profile of infants hospitalized for more than 1 day after birth (n = 557, 70.8%) was compared to the profile of infants without this hospital exposure with the Mann–Whitney U-test.

**Results:** Newborn exposure to the hospital environment for more than one day was associated with higher fecal abundance, at infant age 3–4 months, of microbes belonging to the Lachnospiraceae family (p = 0.041). Moreover, in a comparison analysis restricted to vaginally delivered and not treated with antibiotic at birth (both directly and indirectly), the Pseudomonadaceae (p = 0.04) and genus *Pseudomonas* (p = 0.027) were more abundant in the gut of infants with extended hospital stays after birth. This dysbiosis was evident in both vaginally and caesarean-delivered infants.

**Conclusion:** This study highlights the association between extended exposure to the hospital microbial environment after birth and changes to infant gut microbial composition, including greater colonization with hospital-acquired pathogens, within the first 3 months of life. The impact of these compositional changes on the development of gut immunity and atopic disease later in life requires further study.

**Acknowledgements:** Sincere thanks to the CHILD research team and all study families. This research is supported by CIHR, AllerGen, University of Manitoba, University of Toronto and University of Alberta.

**References**

1. Gravel D, Matlow A, Ofner-Agostini M, Loeb M, Johnston L, Bryce E, Sample M, Roth V, Goldman C, Taylor G. A point prevalence survey of health care associated infections in pediatric populations in major Canadian acute care hospitals. Am J Infect Control. 2007;35:157–62.

2. Canadian Institute of Health Information. https://secure.cihi.ca/free_products/Costs_Report_06_Eng.pdf. Accessed 24 Mar 2016.

## A34 Investigating differential expression patterns of complement system-related genes in individuals with allergic asthma

### ChenXi Yang^1,2,3^, Amrit Singh^1,2^, Casey P. Shannon^1,2^, YoungWoong Kim^1,2^, Gail M. Gauvreau^4^, Edward M. Conway^3^, Scott J. Tebbutt^1,2,5^

#### ^1^James Hogg Research Centre for Heart Lung Innovation, St. Paul’s Hospital, The University of British Columbia, Vancouver, BC, Canada, ^2^Prevention of Organ Failure (PROOF) Centre of Excellence, Vancouver, BC, Canada, ^3^Centre for Blood Research, University of British Columbia, Vancouver, BC, Canada, ^4^Department of Medicine, McMaster University, Hamilton, ON, Canada, ^5^Department of Medicine, Division of Respiratory Medicine, University of British Columbia, Vancouver, BC, Canada

##### **Correspondence:** ChenXi Yang - yolanda.yang@hli.ubc.ca

*Allergy, Asthma & Clinical Immunology* 2016, **12(Suppl 2)**:A34

**Background:** Allergic asthma is a chronic inflammatory disorder of the airways, often characterized by biphasic airway contraction in response to controlled inhalation of allergens. The molecular mechanisms underlying the difference between an early phase responder (ER), who experiences an isolated bronchoconstriction within minutes following the allergen exposure, and a dual phase responder (DR), who additionally experiences a chronic bronchoconstriction several hours after the initial exposure, remain elusive. As a danger sensing component of the innate immune system, activation of complement system is strongly associated with the disease [1]. In this study, the effect of complement system-related genes on allergic asthma was investigated and validated. We hypothesize that complement system-related genes are differentially expressed in ERs and DRs.

**Methods:** Peripheral whole blood samples were collected using PAXgene Blood RNA tubes from mild asthmatic and non-asthmatic subjects. 8 complement system-related genes were previously identified in a discovery cohort of 29 subjects with RNA-sequencing. NanoString nCounter Elements assays were applied to the same discovery cohort and additional subjects (n = 42, ER = 16, DR = 26), as well as another cohort (n = 64, ER = 4, DR = 52, Control = 8) to validate the findings. Differential expression analysis was used to identify significant genes.

**Results:** With a Benjamini-Hochberg false discovery rate cutoff of 0.1, SERPINA1, PLAUR and C5AR1 were down-regulated in DRs of the discovery cohort and additional subjects. No significant differences in gene expression were found between ERs and DRs in the validation cohort; however, F13A1, CD46, C5AR1, SERPINA1, PLAUR, CD59 and C3AR1 were up-regulated in asthmatic individuals in comparison to non-asthmatic controls.

**Conclusions:** The complement system plays an important role in allergic asthma, as well as in the late phase response of allergic asthma. More ERs are needed to further validate the results.

**Acknowledgements:** We would like to thank our research participants and the staff and investigators of the AllerGen NCE Clinical Investigator Collaborative. This study was funded by AllerGen NCE, BC Lung Association, and Mitacs.

**Reference**

1. Zhang X, Kohl J. A complex role of complement in allergic asthma. Expert Rev Clin Immunol. 2010;6(2):269–77

## A35 Investigating systemic immune responses in peripheral blood of cat-allergic people under the nasal allergen challenge model

### Young Woong Kim^1,2,3^, Casey P. Shannon^3^, Amrit Singh^1,2,3^, Anne K. Ellis^4,5^, Helen Neighbour^6,7^, Mark Larché^6,7^, Scott J Tebbutt^1,2,3,8^

#### ^1^Experimental Medicine, University of British Columbia, Vancouver, BC, Canada, ^2^James Hogg Centre for Heart Lung Innovation, St. Paul’s Hospital, Vancouver, BC, Canada, ^3^Prevention of Organ Failure (PROOF) Centre of Excellence, Vancouver, BC, Canada, ^4^Departments of Medicine and Biomedical & Molecular Science, Queen’s University, Kingston, ON, Canada, ^5^Allergy Research Unit, Kingston General Hospital, Kingston, ON, Canada, ^6^Department of Medicine, McMaster University, Hamilton, ON, Canada, ^7^Firestone Institute for Respiratory Health, McMaster University, Hamilton, ON, Canada, ^8^Department of Medicine (Division of Respiratory Medicine), University of British Columbia, Vancouver, BC, Canada

##### **Correspondence:** Young Woong Kim - youngwoong.kim@hli.ubc.ca

*Allergy, Asthma & Clinical Immunology* 2016, **12(Suppl 2)**:A35

**Background:** Allergic rhinitis (AR), a prevalent disease Worldwide, is an IgE-mediated inflammatory condition of the nasal mucosa induced after allergen exposure [1]. Cat allergy affects 10–15% of patients with allergic rhinitis and/or asthma [2]. The standardized nasal allergen (NAC) model may allow us to investigate into AR pathophysiology with biological sampling [3].

**Methods:** In this study, 19 participants with a clinical history of allergy to cats underwent NAC. Clinical symptom changes post NAC were measured by peak nasal inspiratory flow (PNIF) for assessing nasal patency and total nasal symptom score (TNSS) for participants’ symptom recognition. Whole blood was collected into PAXgene tubes at baseline and post NAC (1, 2 and 6 h). The PAXgene blood lysates were applied to profile 770 immune genes using the nanoString nCounter PanCancer Immune Profiling Panel. The profiled gene expression data was normalized by nSolver (ver. 2.6). The statistical analyses of the complete blood count (CBC) data and the gene expression data were performed using the R statistical computing program {Packages: limma (ver. 3.26.8), nlme (ver. 3.1-125), Mfuzz (ver. 2.30.0)}.

**Results:** The clinical symptom changes in PNIF and TNSS demonstrated that the NAC had triggered AR response in participants. The comparisons in the CBC and the gene expression data were implemented both at each time point post NAC by comparing to baseline and over time points to identify continuously changed variable. The numbers of lymphocytes and monocytes were significantly changed at 6 h post NAC compared to baseline and increased over time (p value <0.05). Meanwhile, the number of neutrophils was significantly changed at comparison between each time point (1, 2 and 6 h) post NAC and baseline (p value <0.05) but not over time. 202 immune genes were significantly differentially expressed over time points (p value <0.05), and the gene expression patterns over time were clustered into 8 groups using the fuzzy c-means algorithm.

**Conclusions:** Investigations in peripheral blood collected during NAC identified significant changes in blood cell counts and the whole blood transcriptome in parallel with the onset of AR. Although the investigation wasn’t able to cover the whole immune response, it is a cross-section that may provide biomarkers of AR pathophysiology which could be applied to the investigation of mechanisms of action of allergen immunotherapy.

**References**

1. Pawankar R, et al. Allergic Rhinitis and Its Impact on Asthma in Asia Pacific and the ARIA Update 2008. World Allergy Organ J. 2012;5(Suppl 3):S212–7.

2. Patel D, et al. Fel d 1–derived peptide antigen desensitization shows a persistent treatment effect 1 year after the start of dosing: A randomized, placebo-controlled study. J Allergy Clin Immunol. 2013;131(1):103–9.

3. Ellis AK, et al. The Allergic Rhinitis—Clinical Investigator Collaborative (AR-CIC): nasal allergen challenge protocol optimization for studying AR pathophysiology and evaluating novel therapies. Allergy Asthma Clin Immunol. 2015;11(1):16.

